# Nasal Epithelial Immune Signatures Are Associated With Age‐Dependent Asthma Trajectories in Early Life

**DOI:** 10.1111/all.70477

**Published:** 2026-09-06

**Authors:** Constanze A. Jakwerth, Sabina Illi, Helen Charles, Madlen Oelsner, Amr A. Gabr, Ulrich M. Zissler, Anna Erb, Martin Feuerherd, Felix Drost, Maren Büttner, Benjamin Schubert, Nicole Maison, Jimmy Omony, Markus Weckmann, Ruth Grychtol, Anna‐Maria Dittrich, Svenja Foth, Chrysanthi Skevaki, Tobias Trojan, Silke van Koningsbruggen‐Rietschel, Gesine Hansen, Thomas Bahmer, Klaus F. Rabe, Folke Brinkmann, Matthias Volkmar Kopp, Ulrike Protzer, Adam M. Chaker, Bianca Schaub, Erika von Mutius, Carsten B. Schmidt‐Weber, the ALLIANCE Study Group as part of the German Center for Lung Research (DZL), Mira Bürk, Sybille Contento, Markus Ege, Kristina Gottschau, Silvia Gschwendtner, Alexander Hose, Lena Lagally, Michael Schloter, Lena Ullemeyer, Esther Zeitlmann, Mustafa Abdo, Heike Biller, Karoline I. Gaede, Clara Haug, Nikolas Jacobs, Anne‐Marie Kirsten, Inke R. König, Lea Kronziel, Gyde Nissen, Frauke Pedersen, Loana Pennerd, Isabell Ricklefs, Vera Veith, Henrik Watz, Marie Bickes, Mifflin‐Rae Calvero, David S. DeLuca, Svenja Gaedcke, Anika Habener, Christine Happle, Adan Chari Jirmo, Bin Liu, Lennart Riemann, Nicolaus Schwerk, Inga Jerrentrup, Harald Renz, Stefanie Weber, Miguel A. Alejandre Alcazar, Samira Blau, Ernst Rietschel, Jan‐Christoph Thomassen

**Affiliations:** ^1^ School of Medicine and Health, Center of Allergy and Environment (ZAUM) Technical University of Munich Munich Germany; ^2^ Helmholtz Munich Munich Germany; ^3^ Member of the German Center for Lung Research (DZL) Germany; ^4^ Institute for Asthma and Allergy Prevention (IAP), Helmholtz Munich, German Research Center for Environmental Health (GmbH) Munich Germany; ^5^ Comprehensive Pneumology Center – Munich (CPC‐M) Munich Germany; ^6^ Institute of Virology, School of Medicine and Health Technical University of Munich Munich Germany; ^7^ German Center for Infection Research (DZIF), Munich Partner Site Munich Germany; ^8^ Institute of Computational Biology Helmholtz Munich—German Research Center for Environmental Health Neuherberg Germany; ^9^ School of Life Sciences Weihenstephan Technical University of Munich Munich Germany; ^10^ Department of Mathematics Technical University of Munich Garching bei München Germany; ^11^ Department of Pulmonary and Allergy, Dr von Hauner Children's Hospital, University Children's Hospital Ludwig Maximilians University Munich Germany; ^12^ Division of Epigenetics in Chronic Lung Disease, Priority Area Chronic Lung Diseases Research Center Borstel‐Leibniz Lung Center Borstel Germany; ^13^ Division of Pediatric Pneumology and Allergology, University Children's Hospital Lübeck University Hospital Schleswig‐Holstein Lübeck Germany; ^14^ Airway Research Center North (ARCN) Germany; ^15^ Department of Paediatric Pneumology, Allergology and Neonatology Hannover Medical School Hannover Germany; ^16^ Biomedical Research in Endstage and Obstructive Lung Disease Hannover (BREATH) Hannover Germany; ^17^ Universities of Giessen and Marburg Lung Center (UGMLC), Philipps University Marburg Marburg Germany; ^18^ University Children's Hospital Marburg, University of Marburg Marburg Germany; ^19^ Institute of Laboratory Medicine and Pathobiochemistry, Molecular Diagnostics University of Marburg Marburg Germany; ^20^ University of Gießen, Marburg Lung Center (UGMLC) Giessen Germany; ^21^ Pediatric Pulmonology and Allergology, Department of Pediatrics, Faculty of Medicine and University Hospital Cologne University of Cologne Cologne Germany; ^22^ Cluster of Excellence RESIST (EXC 2155) Hannover Medical School Hannover Germany; ^23^ Internal Medicine Department I University Hospital Schleswig‐Holstein ‐ Campus Kiel Kiel Germany; ^24^ LungenClinic Grosshansdorf GmbH and Medical Clinics Christian Albrechts University Kiel Germany; ^25^ University Children's Hospital, Inselspital, Bern University Hospital University of Bern Bern Switzerland; ^26^ Department of Otorhinolaryngology, TUM School of Medicine and Health, Klinikum rechts der Isar Technical University of Munich Munich Germany; ^27^ German Center for Child & Adolescent Health (DZKJ) Dr v Hauner Children's Hospital, LMU Munich Munich Germany

**Keywords:** airway epithelium, asthma, early life wheeze, epithelial immunity, nasal transcriptomics, type‐2 inflammation

## Abstract

**Background:**

Preschool wheeze is heterogeneous and only a subset of children progresses to persistent asthma. We hypothesized that early‐life wheeze reflects divergent airway epithelial maturation trajectories associated with distinct mucosal immune programs.

**Methods:**

Nasal epithelial transcriptomes from 265 children and young adults (79 healthy, 81 wheezers, 105 asthmatics; median age 10.4 [1.1–20.3] years) from the All Age Asthma Cohort (ALLIANCE) cohort were analyzed using a hypothesis‐driven candidate approach based on predefined cytokine axes. Transcriptional signatures were mapped to epithelial cells by scRNA‐sequencing. Asthma‐outcome associations were assessed in age‐ and sensitization‐adjusted multivariate models.

**Results:**

The candidate approach identified three antagonistic epithelial immune programs: an interferon‐associated (E1; IDO1, CSF3, CCL20), a type‐2‐associated (E2; POSTN, CCL26, CST1), and a type‐17‐associated program (E3; IL36G, KLK7, KRTDAP). These programs were detected across basal, ciliated, and secretory epithelial cells. In early childhood (1–3 years), wheezers predominantly exhibited E1/E3‐dominant epithelial profiles (*n* = 36). Age‐group‐specific comparisons showed higher E2 and lower E1/E3 expression in wheezers aged 4–6 years (*n* = 38) than in those aged 1–3 years (*n* = 36). E2 dominance correlated with IgE, FeNO, and eosinophilia (*p* < 0.01). In multivariate models assessing asthma outcome at age 6, E1 expression was inversely associated with asthma persistence (OR = 0.15, *p* < 0.05), whereas E3 expression was associated with disease progression (OR = 4.93, *p* < 0.05). The combined epithelial signature discriminated asthma outcomes with high accuracy (AUC = 0.90), independent of allergic sensitization.

**Conclusion:**

Early‐life wheeze reflects distinct airway epithelial immune programs characterized by age‐associated patterns in epithelial immune activity. Nasal epithelial transcriptional signatures capture molecular trajectories associated with asthma persistence and may enable early risk stratification.

**Trial Registration:**

All‐Age‐Asthma (ALLIANCE) cohort: clinicaltrials.gov: NCT02496468; adult arm: NCT02419274

## Graphical abstract

Nasal epithelial transcriptomics identified three cytokine‐associated epithelial immune programs linked to childhood asthma phenotypes. Age‐associated epithelial polarization shifted from IFN/type‐17‐associated toward type‐2‐associated programs after 3 years of age. Early epithelial signatures were associated with persistent asthma and stratified risk with high accuracy (AUC = 0.90). ALLIANCE, ALL‐Age‐Asthma; AUC, area under the curve; CCL, C‐C motif chemokine ligand; CSF3, colony stimulating factor 3; CST1, cystatin SN; IDO1, indoleamine 2,3‐dioxygenase 1; IFN, interferon; IL, interleukin; KLK7, kallikrein‐related peptidase 7; KRTDAP, keratinocyte differentiation associated protein; OR, odds ratio; POSTN, periostin.

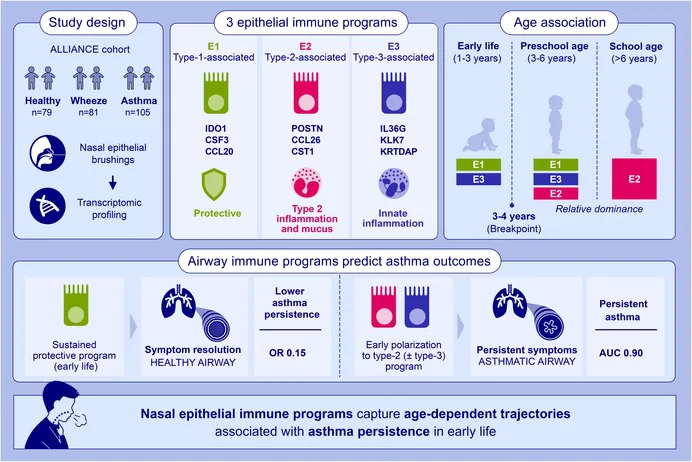

## 1 Introduction

Asthma affects more than 300 million individuals worldwide. Airway obstruction in asthma is often triggered by viral infections or allergens that impair epithelial integrity and amplify mucosal inflammation [1, 2, 3, 4]. The airway epithelium is a dynamic physical and immunological barrier essential for host defense and homeostasis [5, 6]. Differentiation into specialized epithelial cell types supports barrier integrity and tailored responses to environmental insults and infection [5]. In asthma, epithelial differentiation and barrier function are impaired, and type‐2 inflammatory pathways become chronically activated through persistent Th2‐cell, mast‐cell, and eosinophil infiltration [7, 8, 9].

Beyond serving as a passive barrier, epithelial cells integrate cytokine signals that durably imprint immune programs in the mucosa. Distinct cytokine milieux drive polarized epithelial phenotypes: interferon (IFN)‐γ‐driven (type‐1), IL‐4/IL‐13‐driven (type‐2), and IL‐17‐dominated (type‐3), governing antiviral defense, mucus production, and neutrophilic inflammation, respectively [10, 11, 12]. Although these cytokine‐imprinted epithelial programs influence barrier integrity and immune cell recruitment, their establishment in vivo and relevance to asthma development remain unclear. Defining these epithelial immune programs in human airways may reveal early molecular trajectories of asthma and identify biomarkers accessible by noninvasive sampling.

Using nasal transcriptomics from the longitudinal ALLIANCE asthma cohort, we applied a hypothesis‐driven approach focused on predefined type‐1, type‐2, and type‐3 cytokine axes and identified cytokine‐linked epithelial response programs—E1 (type‐1), E2 (type‐2), and E3 (type‐3)—that mirror mucosal cytokine networks and associate with divergent asthma trajectories in early childhood. Building on prior evidence for IFN‐γ‐ and IL‐4‐mediated epithelial polarization [11, 12], we investigated the precursors of this polarization and observed a previously unrecognized type‐3‐associated epithelial program in this nasal transcriptomic context. These findings establish nasal epithelial immune states as noninvasive markers of mucosal polarization and highlight imprinting mechanisms relevant to airway homeostasis.

## 2 Results

### 2.1 Identification of Three Major Nasal Immunophenotypes

To assess the immunological dynamics of the airway epithelial cells at the site of inflammation, we analyzed the nasal transcriptome from 265 genetically unrelated children in the pediatric arm of the ALLIANCE cohort, classified into “preschool wheeze” (“wheezers,” WH, ≤ 6 years, median age 4.3), “asthma” (AA, > 6 years, median age 13.1), and “healthy” (HC, median age 12.8) (Table 1). To enrich for epithelial mediators and reduce noise from noninformative transcripts, transcript selection was guided by Gene Ontology annotations related to extracellular localization or cytokine/chemokine activity (Section 4). Candidate transcripts ranked by Spearman correlation with predefined type‐1, type‐2, and type‐3 cytokine seed variables identified three epithelial immune programs (‘nasotypes’): E1 (interferon‐associated; *IDO1, CSF3, CCL20, CXCL5, IFNG, OAS2*), E2 (IL‐4/IL‐13‐associated; *POSTN, CCL26, FETUB, CST1, CST2, CPA3*), and E3 (IL‐17‐linked; *IL36G, KLK7, KRTDAP, RNASE7, IL6, IL24*) (Figure 1A, Tables [Link], [Link] and S31). To confirm that the identified nasotypes reflect cytokine‐imprinted epithelial programs, samples were stratified into quintiles based on the predefined E1, E2, and E3 seed variables: *IFNG*, mean (*IL4*, *IL13*), and mean (*IL17A*, *IL36G*), respectively. All 18 predefined E1‐, E2‐, and E3‐associated genes showed significant positive trends across increasing quintiles of their respective cytokine seed variables, with moderate‐to‐very‐strong monotonic associations (Spearman's *ρ* = 0.36–0.98; one‐sided Jonckheere‐Terpstra tests, all BH‐adjusted *p* ≤ 3.39 × 10^−9^; Figure 1B–D, Table S3). In differentiated airway epithelial cultures stimulated with IFN‐γ, IL‐4, or IL‐17A + IL‐36γ, each cytokine reproduced the expected transcriptional program, consistent with prior observations of IFN‐γ‐ and IL‐4‐induced epithelial programming [11]. Although derived from nonasthmatic bronchial cells, these cultures recapitulated the polarization pattern observed ex vivo. E1 and E2 markers showed reciprocal expression, supporting antagonistic regulation between type‐1 and type‐2 epithelial immunity.

**TABLE 1:** Subject characteristics of healthy, wheezing, and asthmatic children of the ALLIANCE cohort (DZL).

			Healthy < 6 years		Wheeze		Healthy ≥ 6 years		Asthma		
			*N* = 12		*N* = 81		*N* = 67		*N* = 105		
Male gender		*N* (%)	12	7 (58.3%)	81	54 (66.7%)	67	25 (37.3%)	105	71 (67.6%)	*^,§^
Age (years)		Mean (SD)	12	3.64 (1.38)	79	4.34 (1.74)	67	13.1 (2.91)	105	12.5 (3.17)	*^,†,‡^
		Median [Min, Max]		3.15 [1.70, 5.90]		4.30 [1.10, 7.90]		13.2 [6.0, 20.3]		13.1 [6.30, 18.8]	
Eosinophils (cells·μL^−1^)		Mean (SD)	11	303.1 (209.4)	75	403.7 (469.1)	64	220.0 (189.6)	101	454.9 (345.5)	*^,§,†^
		Median [Min, Max]		240.3 [76.0, 700.8]		292.0 [0.0, 3132.0]		157.7 [0.0, 757.0]		380.5 [0.00, 1856.0]	
Neutrophils (cells·μL^−1^)		Mean (SD)	11	3752 (1257)	75	3917 (1867)	66	3608 (1277)	103	3497 (1351)	
		Median [Min, Max]		3572 [2331, 6032]		3634 [774.1, 10890]		3314 [1227, 6800]		3341 [1433, 10,050]	
BMI (kg/m^2^)		Mean (SD)	12	16.7 (1.35)	79	16.3 (2.00)	67	19.8 (3.41)	105	21.2 (5.53)	*^,†,‡^
		Median [Min, Max]		16.8 [14.2, 18.8]		15.9 [11.6, 23.4]		19.3 [13.9, 31.1]		20.4 [12.7, 43.3]	
FeNO (ppb)		Mean (SD)		NA	18	12.8 (12.6)	53	19.4 (20.1)	87	24.0 (22.9)	*^,†,‡^
		Median [Min, Max]				8.91 [1.25, 49.8]		11.0 [1.66, 76.0]		16.5 [2.52, 118.4]	
FEV1 (*z*‐score)		Mean (SD)		NA		NA	64	0.11 (0.99)	105	−0.29 (1.02)	^§^
		Median [Min, Max]						0.12 [−1.80, 3.06]		−0.37 [−2.38, 2.69]	
FVC (*z*‐score)		Mean (SD)		NA		NA	64	−0.06 (1.03)	105	0.15 (0.95)	
		Median [Min, Max]						−0.0 [−2.49, 2.0]		0.19 [−2.45, 2.04]	
FEV1/FVC (*z*‐score)		Mean (SD)		NA		NA	64	0.44 (1.98)	105	−0.70 (1.06)	^§^
		Median [Min, Max]						0.08 [−1.97, 13.82]		−0.68 [−2.90, 1.93]	
Any exacerbation past 12 months		*N* (%)		NA	79	38 (48.1%)		NA	104	19 (18.3%)	*^,†^
ICS use past 12 months		*N* (%)		NA	78	43 (55.1%)		NA	103	44 (42.7%)	
GINA	Controlled	*N* (%)		NA	78	41 (52.6%)		NA	105	59 (56.2%)	^†^
	Partially controlled					21 (26.9%)				38 (36.2%)	
	Uncontrolled					16 (20.5%)				8 (7.6%)	
Parental hay fever		*N* (%)	12	7 (58.3%)	81	40 (49.4%)	66	29 (43.9%)	103	59 (57.3%)	
Parental eczema		*N* (%)	12	3 (25.0%)	80	20 (25.0%)	65	9 (13.8%)	104	31 (29.8%)	^§^
Parental asthma		*N* (%)	12	3 (25.0%)	81	32 (39.5%)	66	12 (18.2%)	103	45 (43.7%)	*^,§^
Atopic sensitization (RAST II)		*N* (%)	11	1 (9.1%)	71	35 (49.3%)	64	31 (48.4%)	102	81 (79.4%)	*^,&,§,†,‡^
ETS (≥ 10/day)		*N* (%)	12	1 (8.3%)	77	10 (13.0%)	63	9 (14.3%)	102	20 (19.6%)	
Maternal smoke exposure (pregnancy)		*N* (%)	12	0 (0.0%)	81	9 (11.1%)	67	5 (7.5%)	105	12 (11.4%)	
Symptoms of ear/nose/throat infection^$^		*N* (%)	12	1 (8.3%)	79	15 (19.0%)	67	5 (7.5%)	105	17 (16.2%)	

*Note:* Data depict the frequencies of the three subject groups: Healthy, wheeze, and asthma of the pediatric arm of the ALLIANCE cohort. Two‐sided Mann–Whitney‐*U*/Chi^2^‐Test: *p < 0.05 overall, ^&^ *p* < 0.05 within < 6 years, ^§^ *p* < 0.05 within ≥ 6 years, ^†^ *p* < 0.05 within diseased, ^‡^ *p* < 0.05 within healthy; ^$^at time of clinical examination.

**FIGURE 1:**
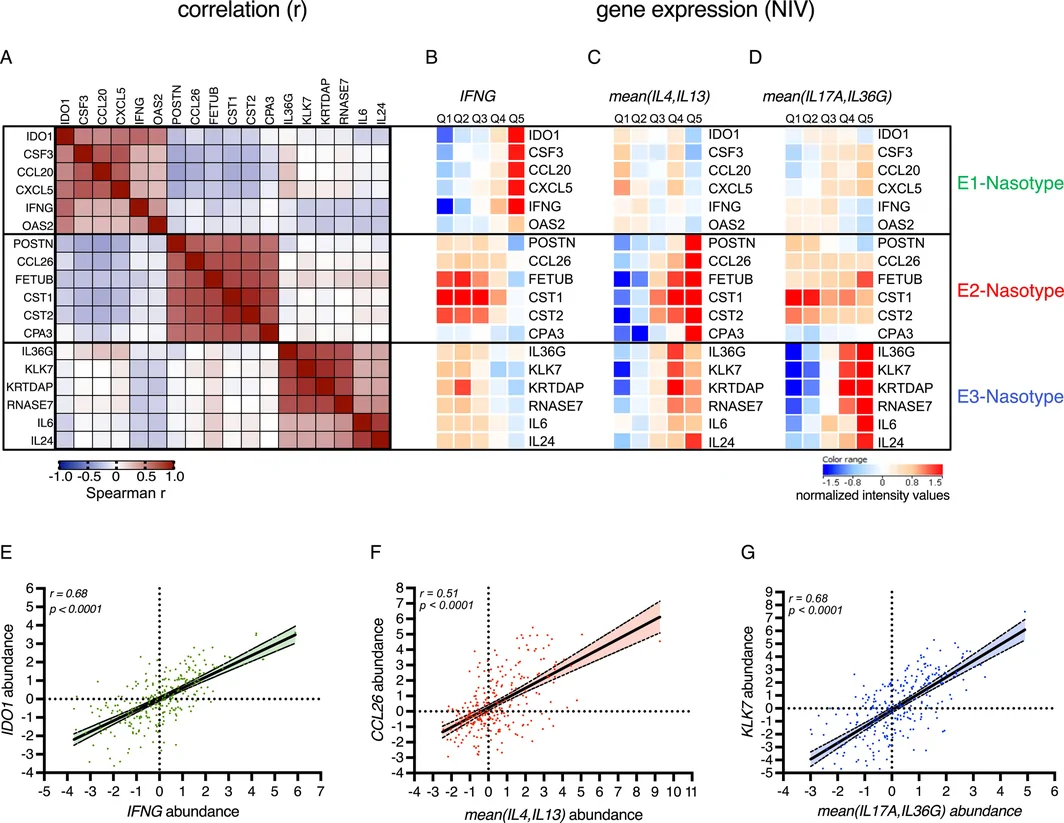
Nasal gene expression clusters in three distinct epithelial nasotypes E1–E3. Correlation matrix (A) and gene expression intensity of genes with secreted gene products (Section 4), measured in nasal transcriptomics from all children (*N =* 265) with and without asthma (B–D). Candidate transcripts were ranked by Spearman correlation with predefined cytokine seed variables representing type‐1, type‐2, and type‐3 immunity, and final module genes were selected based on both seed association and within‐module positive pairwise correlations. Spearman's rank correlation value r is illustrated in blue for negative and red for positive correlation values for the six representative genes of each epithelial immune module. All 265 genetically unrelated independent children were stratified into quintiles based on IFNG expression for E1 (B), *mean(IL4, IL13)* expression for E2 (C), and *mean(IL17A, IL36G)* expression for E3 (D) (Q1–Q5; *N* = 53 per quintile). Increasing trends across quintiles were assessed using one‐sided Jonckheere‐Terpstra tests with asymptotic *p* values and Benjamini–Hochberg correction within each nasotype module. Red boxes indicate genes with high abundance, white boxes with medium abundance, and blue boxes with low abundance. Corresponding gene symbols are listed on the right side of the panel. Gene expression intensity is represented by a color scale from −1.5 to 1.5 of normalized intensity values (NIV). For visual guidance, genes are ordered by nasotype module (E1–E3); color intensity reflects within‐cohort Spearman correlation coefficients. Spearman correlation of (E) type‐1 model gene *IDO1* gene expression with *IFNG* expression, (F) type‐2 model gene *CCL26* gene expression with *mean(IL4, IL13)* expression, and (G) type‐3 model gene *KLK7* gene expression with *mean(IL17A, IL36G)* expression (*N* = 265). The graph depicts mean simple linear regression with a 95% confidence interval. All graphs show data from all children included in the ALLIANCE cohort (*N* = 265).

### 2.2 Independent Replication of Nasotype Gene Modules Across External Cohorts

To assess the reproducibility of nasal immune states across age groups, we replicated the analysis in independent cohorts. In the adult arm of the ALLIANCE cohort (*N* = 174; one sample per subject; Figure S2), the E1‐E3 pattern mirrored the pediatric profile, with two age‐related distinctions (Figure S4A, Tables S4 and S5): (i) the adult E3 state subdivided into *IL36G*‐ and *IL6*‐dominant subclusters, and (ii) correlations between *IFNG*, *IDO1*, and *CSF3* shifted toward the *IL6*‐associated E3 subtype in participants > 65 years. In the adult ALLIANCE cohort, E3‐associated expression increased with age, particularly in participants > 65 years, whereas E2‐associated expression was reduced. In the pediatric cohort, strong E3‐associated correlations were also observed in young healthy children and preschool wheezers aged 1‐3 years.

To address potential circularity and cohort‐specific overfitting, we assessed whether the nasotype gene modules defined in ALLIANCE were preserved in three independent external cohorts: the pediatric URECA cohort (*N* = 353; GSE145505 [13]), the GRASS allergic rhinitis cohort (59 patients, 256 samples; GSE206151 [14]), and the CatEEC cohort (19 patients, 256 samples; GSE129748 [15]). Despite cohort‐specific heterogeneity in age, disease context and platform, the within‐module gene–gene correlation structure of E1 and E2 was robustly observed across datasets. The E3 module was also detectable across replication cohorts, although with lower within‐module correlation strength than in the ALLIANCE cohort (Figure S3, Table 2, Tables S6–S8).

**TABLE 2:** Reproducibility of E1–E3 nasotype within‐module gene–gene correlation patterns across independent nasal transcriptome cohorts.

Cohort/subgroup	E1 module *r* (median [range])	E2 module *r* (median [range])	E3 module *r* (median [range])
ALLIANCE	0.52 [0.33–0.77]	0.78 [0.65–0.96]	0.52 [0.34–0.88]
CatEEC (all)	0.50 [−0.14–0.79]	0.58 [0.38–0.90]	0.37 [−0.02–0.68]
CatEEC group A	0.51 [−0.16–0.85]	0.59 [0.41–0.85]	0.37 [−0.03–0.66]
CatEEC group B	0.43 [−0.14–0.78]	0.64 [0.21–0.91]	0.35 [0.03–0.73]
GRASS (all)	0.42 [0.20–0.70]	0.56 [0.39–0.75]	0.05 [−0.08–0.29]
GRASS SCIT	0.38 [0.11–0.71]	0.62 [0.37–0.84]	0.13 [−0.12–0.30]
GRASS SLIT	0.53 [0.20–0.72]	0.49 [0.19–0.74]	−0.01 [−0.14–0.39]
URECA (all)	0.54 [0.26–0.80]	0.71 [0.57–0.87]	0.13 [0.02–0.53]
URECA asthma at age 10	0.55 [0.33–0.81]	0.78 [0.53–0.95]	0.22 [−0.07–0.49]
URECA no asthma at age 10	0.52 [0.20–0.79]	0.67 [0.57–0.82]	0.12 [0.02–0.61]

*Note:* Spearman correlation coefficients summarizing within‐module gene–gene correlations for the E1, E2, and E3 nasotype modules across the external cohorts URECA, GRASS, and CatEEC. Values represent median Spearman correlation coefficients (*r*) summarizing within‐module gene–gene correlations for the E1, E2, and E3 nasotype gene sets. Ranges indicate minimum‐maximum correlation coefficients across gene pairs within each module. *N*_pairs = 15 gene pairs per module.

### 2.3 scRNA‐Seq Identifies Cytokine‐Associated Epithelial Response Programs

To test whether airway epithelial cells can express nasotype‐associated transcriptional programs under defined cytokine conditions, we analyzed single‐cell RNA‐seq data from primary human bronchial epithelial cells (NHBEs) differentiated at the air‐liquid interface (ALI) in vitro under control or IL‐4‐treated conditions to model type‐2‐primed epithelium. Louvain clustering identified canonical epithelial subsets, including basal, club, secretory, and multiciliated cells, as well as rare tuft, ionocyte, and pulmonary neuroendocrine cell (PNECs) populations (Figure 2A, Tables S9–S13). Cluster identities were assigned based on canonical epithelial marker genes (basal, secretory, multiciliated, club, goblet; Figure S4). Cells were scored using nasotype marker sets after excluding immune‐associated transcripts (*IFNG*, *CPA3*) to focus on epithelial expression (Figure 2B, Figure S5). The remaining nasotype‐associated genes were detected across multiple epithelial populations and cell states (Figure S4). These transcriptional programs were not restricted to a single epithelial subset: E1‐like profiles were detected in basal, club, secretory, and multiciliated cells; E2‐like profiles additionally in tuft and PNEC cells; and E3‐like profiles in basal, club, and secretory cells (Figure 2C). IL‐4 exposure induced pronounced structural and cellular remodeling of ALI cultures (Figure S4), with mucus‐rich crypt‐like regions and marked expansion of secretory E2‐cells (medium control = 92 vs. IL‐4 = 28,444 cells; Figure 2D,E, Figure S7A–C), whereas E1‐cells predominated under control conditions (medium = 1293 vs. IL‐4 = 107 cells). Developmental trajectory analysis (Figure S5D–L) indicated that epithelial cells expressing E1‐ and E3‐associated transcriptional programs span multiple differentiation stages, including basal/club and secretory compartments, whereas cells expressing E2‐associated programs were preferentially aligned with IL‐4‐skewed secretory trajectories. In line with this, E2‐program‐expressing cells showed relative enrichment of secretory markers (e.g., *MUC5AC*) and retention of basal‐cell features (*KRT5*, *TP63*), consistent with delayed or altered terminal differentiation (Figure S5M–N). In contrast, E3‐program‐expressing cells were enriched for barrier‐associated gene expression, whereas E2‐program‐expressing cells showed reduced enrichment of barrier integrity pathways (Figure S6J–M). Together, these observations support that nasotype‐associated programs reflect cytokine‐imprinted epithelial response programs linked to differentiation and barrier function, rather than discrete epithelial lineages.

**FIGURE 2:**
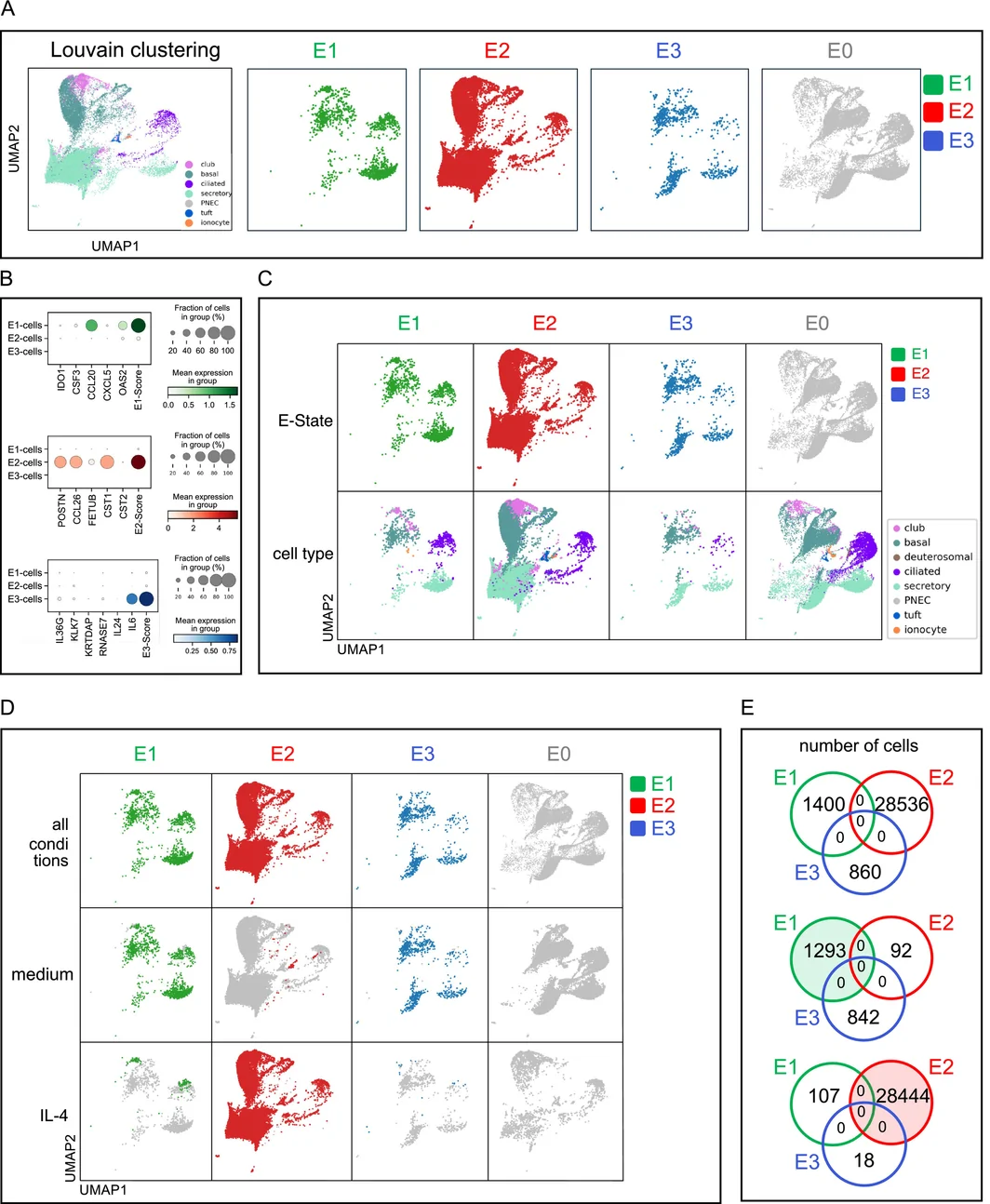
Nasotype‐associated epithelial response programs display distinct expression profiles across epithelial cell types. (A) Single‐cell mRNA‐sequencing analysis of primary human epithelial cells (NHBEs) from three genetically independent donors, which were cultured in a 3D organotypic air–liquid interface (ALI) model and differentiated under control or IL‐4‐treated conditions (50 ng/mL, spiked every 48 h from the basal side) for 12 days before reaching complete epithelial differentiation. Data were analyzed using Louvain cluster analysis, with cell identification performed by the index genes E1 (green), E2 (red), and E3 (blue). The cellular identity in the UMAP clustering is shown with the following cell types: Basal cells (dark green), ciliated cells (purple), club cells (pink), secretory cells (turquoise), PNECs (gray), tuft cells (blue), and ionocytes (orange). (B) Dot plot showing mean expression intensity and detection frequency of E1‐ (green), E2‐ (red), and E3‐associated (blue) marker genes. The size of the circles represents the proportion of cells expressing the respective gene, while color intensity reflects mean expression. Gene‐level expression distributions are shown in Figure S4. (C) The specific cellular identity of the E1, E2, and E3 cells is shown along with the following cell types: Basal cells (dark green), ciliated cells (purple), secretory cells (turquoise), PNECs (gray), tuft cells (blue), ionocytes (orange) and club cells (pink). (D) The influence of differentiation under IL‐4 conditions on the E1, E2, and E3 cells is shown according to the same color code. (E) The Venn diagram shows the number of cells in (D) carrying the index genes E1, E2, and E3. scRNA‐seq analyses illustrate the capacity of epithelial cells to express nasotype‐associated transcriptional programs under cytokine stimulation and are not intended to define in vivo epithelial cell states.

### 2.4 Cytokine Stimulation Induces Epithelial E1–E3 Response Programs In Vitro

To determine whether epithelial E1‐E3 programs can be induced in vitro, primary NHBEs were stimulated with IFNγ, IL‐4, or IL‐17A + IL‐36γ and analyzed by qPCR. IFNγ induced E1‐associated genes, IL‐4 induced E2‐associated genes, and IL‐17A + IL‐36γ induced E3‐associated genes in submerged cultures (Figure 3A–C). To further characterize cytokine‐induced epithelial programs at single‐cell resolution, scRNA‐seq was performed on NHBE ALI cultures from five independent donors, including two donors differentiated under IL‐17A + IL‐36γ conditions (Figure 3D–G; Tables S14–S16). IFNγ stimulation predominantly enriched E1‐associated epithelial programs, whereas IL‐4 induced robust E2‐associated transcriptional states. IL‐17A + IL‐36γ induced a more heterogeneous epithelial response with partial enrichment of E1‐ and E3‐associated programs.

**FIGURE 3:**
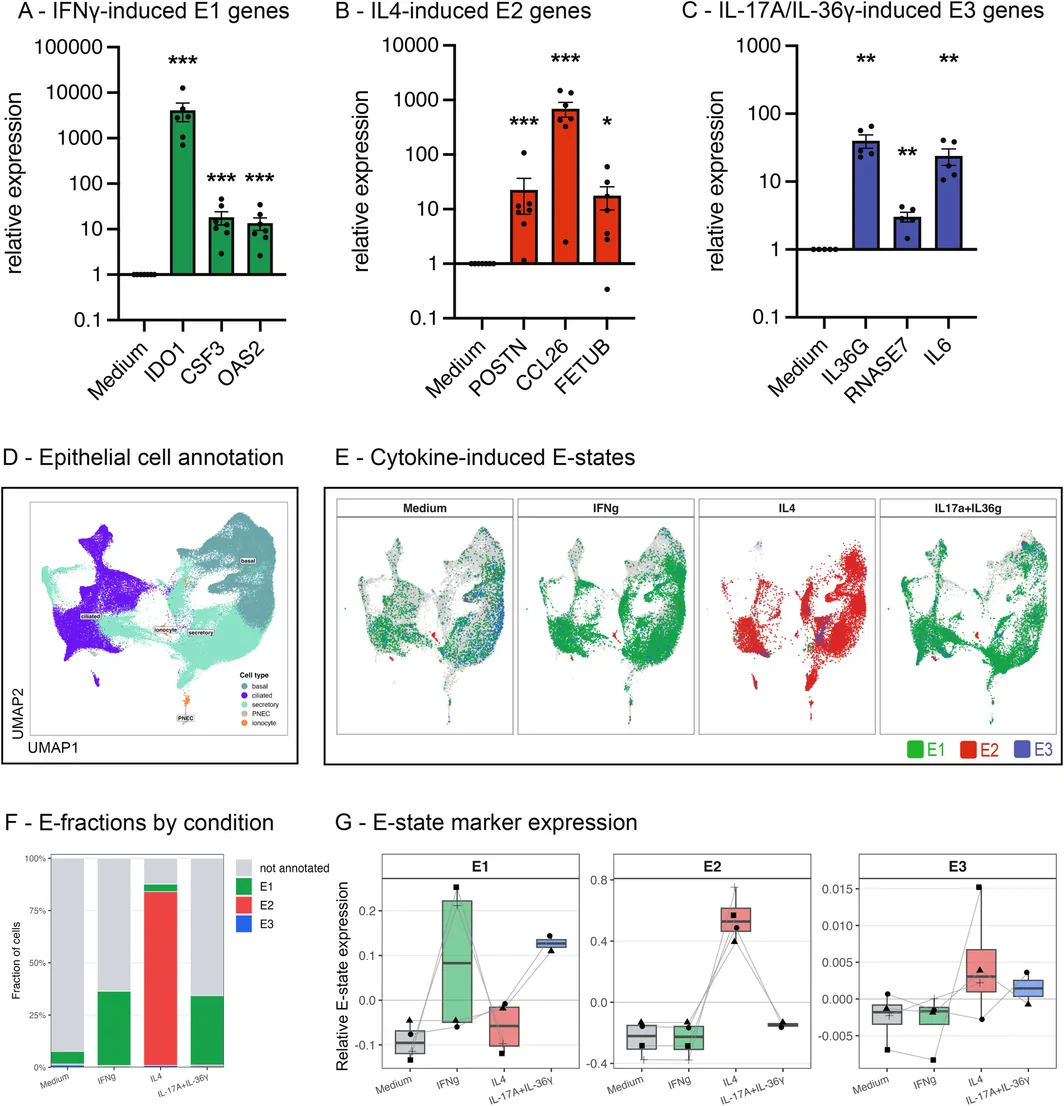
Cytokine‐induced epithelial state programs in primary human bronchial epithelial cells. NHBEs from six genetically independent donors were stimulated in submerged 2D culture with (A) IFN‐γ (100 IU/mL), (B) IL‐4 (50 ng/mL), or (C) IL‐17A (100 ng/mL) plus IL‐36γ (100 ng/mL) for 6 h. Gene expression was quantified by qPCR and is shown as relative expression levels. Asterisks indicate statistical significance (*p < 0.05, **p < 0.01, ***p < 0.001; Mann–Whitney *U* test). For scRNA‐seq, NHBE air‐liquid interface (ALI) cultures from five genetically independent donors were differentiated for 12 days under Medium, IFNγ (100 IU/mL), IL‐4 (50 ng/mL), or IL‐17A (100 ng/mL) plus IL‐36γ (100 ng/mL) conditions to assess inducible epithelial response programs; IL‐17A + IL‐36γ‐treated cultures were available from two donors. (D) UMAP projection of integrated NHBE ALI single‐cell transcriptomes annotated by major epithelial cell types. (E) UMAP projections colored by E‐state assignment under Medium, IFNγ, IL‐4, and IL‐17A + IL‐36γ conditions. IFNγ predominantly induced E1 cells (green), whereas IL‐4 enriched E2 cells (red). IL‐17A + IL‐36γ induced a mixed epithelial response with enrichment of E1‐ and E3‐associated programs and E3 cells (blue); unassigned cells are shown in gray. (F) Relative fractions of E1‐, E2‐, E3‐, and unassigned cells across stimulation conditions. (G) Pseudobulk E‐state marker expression across ALI differentiation conditions. Points and connecting lines represent individual donors.

### 2.5 Epithelial Nasotypes in Healthy, Wheezing, and Asthmatic Children

To explore whether distinct disease states are reflected in nasal immune signatures, we compared nasotype gene expression among healthy, wheezing, and asthmatic children. Healthy children showed a balanced distribution of E1‐, E2‐, and E3‐nasotype expression with slightly reduced E2‐signatures (Figure 4A, Table S17), whereas young wheezers predominantly expressed the E3‐nasotype (Figure 4B, Table S18). Compared with wheezing children, school‐age children with asthma showed dominant E2‐associated gene expression together with lower E3‐associated gene expression (Figure 4C, Tables S19 and S20). Children with a high nasal expression of E1 markers (E1‐high) displayed elevated monocyte and granulocyte markers in preschool years, whereas NK‐ and cytotoxic T‐cell markers were more prominent in children aged ≥ 9 years (Figure S7A). E2‐high children consistently expressed mast‐cell markers across all ages, while E3‐high children showed eosinophil‐, dendritic‐cell‐, macrophage‐, and granulocyte‐associated gene expression. Stratified by disease, mast‐cell markers differed across age groups in children with asthma, but not in healthy children, who showed higher macrophage‐related signatures in early life (Figure S7B). The nasotype matrix also functionally classifies epithelial endotypes in adult‐onset asthma. In the nasal transcriptome analysis of the U‐BIOPRED cohort [16], the “TAC1” gene module (high eosinophils, fractional exhaled nitric oxide (FeNO), serum POSTN) mapped to the E2‐nasotype, whereas the “TAC2” module (high neutrophils, elevated CRP) aligned with E1/E3‐nasotypes (Figure S8, Tables S21 and S22).

**FIGURE 4:**
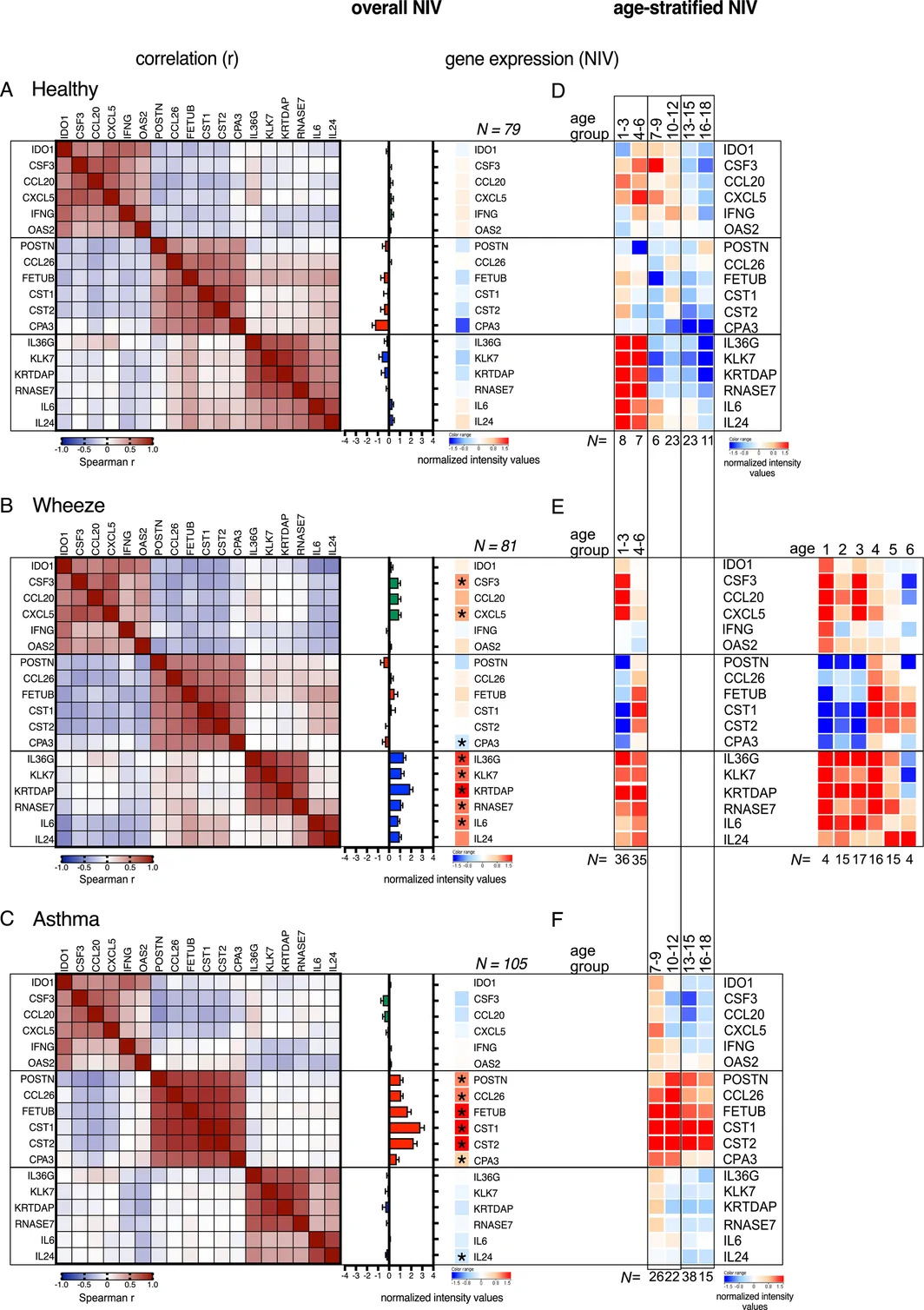
Epithelial E1‐E3 nasotype programs across pediatric airway disease cohorts and age groups. Correlation matrix and gene expression intensity of nasal brushings from (A) healthy children (*N* = 79), (B) wheezers (age 0–6, *N* = 81), and (C) asthma (age 6–18, *N* = 105). Correlation matrices show within‐cohort Spearman rank correlation coefficients for E1–E3 index genes (blue, negative; red, positive correlations). Genes are ordered by E1–E3 nasotype module. Gene expression intensity is shown as normalized intensity values (NIV; −1.5 to 1.5) in corresponding heatmaps and bar plots for E1 (green), E2 (red), and E3 (blue) genes. Red indicates high abundance, white intermediate abundance, and blue low abundance. Asterisks denote statistical significance compared with healthy controls. The left NIV panel summarizes overall nasotype‐associated gene expression within each disease group, whereas the right NIV heatmaps display age‐stratified expression patterns across the indicated age groups. (A–C) Comparison of healthy children (*N* = 79), wheezers aged 0–6 years (*N* = 71), and asthmatic children aged 6–18 years (*N* = 105). (D–F) Age‐stratified analysis across the following age groups: 1–3, 4–6, 7–9, 10–12, 13–15, and 16–18 years. Healthy: 1–3 (*N* = 8), 4–6 (*N* = 7), 7–9 (*N* = 6), 10–12 (*N* = 23), 13–15 (*N* = 23), 16–18 (*N* = 11); Wheeze: 1 year (*N* = 4), 2 years (*N* = 15), 3 years (*N* = 17), 4 years (*N* = 16), 5 years (*N* = 15), 6 years (*N* = 4); Asthma: 7–9 (*N* = 26), 10–12 (*N* = 22), 13–15 (*N* = 38), 16–18 (*N* = 15). Statistical analyses supporting the age‐stratified and disease‐group comparisons. Differential expression was assessed using moderated *t*‐tests with Benjamini‐Hochberg correction (adjusted *p* < 0.05, fold change > 1.5).

### 2.6 Age‐Associated Differences in Epithelial Program Expression

Given the marked nasotype shift observed in school‐age children, we next assessed age‐dependent expression patterns across disease groups. Linear regression models revealed significant correlations between age and nasotype activity, with E1 and E3 showing lower and E2 higher expression across age groups, including a significant age interaction for E2 (Tables S22 and S23). In young healthy children, activation of the E3‐nasotype was predominant, while E1 was only moderately expressed (Figure 4D, Table S24). From school age onwards, no dominant nasotype pattern was detected in healthy subjects. In wheezing children, ages 1–3 were characterized by strong E1‐ and E3‐nasotype expression, accompanied by low E2 abundance (Figure 4E). Wheezers aged 4–6 showed higher E2 expression, together with lower E1 and E3 activity (Table S25). E3‐associated expression was higher in wheezers aged 1–3 years than in those aged 4–6 years (Figure 4E, Table S25). In younger asthmatics, mixed nasotype profiles were observed, but E2‐nasotype expression dominated from age 9 onwards during puberty and adolescence (Figure 4F). Collectively, these data reveal an age‐group‐specific contrast, with E1/E3‐predominance in wheezers aged ≤ 3 years and an E2‐biased pattern in those aged 4–6 years.

### 2.7 Nasotype Association With Clinical Parameters in the Pediatric ALLIANCE Cohort

E2‐nasotype expression correlated with multiple atopy‐related parameters, including peripheral eosinophil counts, total and specific IgE (≥ 0.7 kU/L, RAST class II), the cumulative sum of allergen‐specific IgE values (EuroImmun Allergy assay), and allergen‐triggered wheezing episodes during the past 12 months (pollen, dust, or animal exposure; Figure 5A, Table S26). Clinical symptoms and inhaled corticosteroid (ICS) use were recorded via standardized questionnaires in the longitudinal pediatric ALLIANCE follow‐up. Children with atopy‐related symptoms displayed higher E2‐ and lower E1‐nasotype expression than unaffected peers (Figure 5B–E). E2 activation was associated with animal‐triggered wheeze, eosinophilia, and elevated fractional exhaled nitric oxide (FeNO; low ≤ 20 ppb, intermediate/high > 20 ppb), but not with psychological triggers or ICS use (Figure 5G–J). Linear regression models confirmed E1‐nasotype as negatively associated with FeNO and allergen‐specific IgE (Table S28). At the same time, E2‐nasotype correlated positively with eosinophils, FeNO, and allergic symptoms such as itchy nose and eyes (Table S29). E3‐nasotype showed no consistent associations, except weak links to ICS use, blood neutrophilia, and upper respiratory tract infection symptoms (Table S30). Together, these findings support a close relationship between type‐2‐polarized epithelial programs and allergic sensitization, whereas IFN‐γ and IL‐17‐linked epithelial states are associated with nonatopic, infection‐related airway inflammation.

**FIGURE 5:**
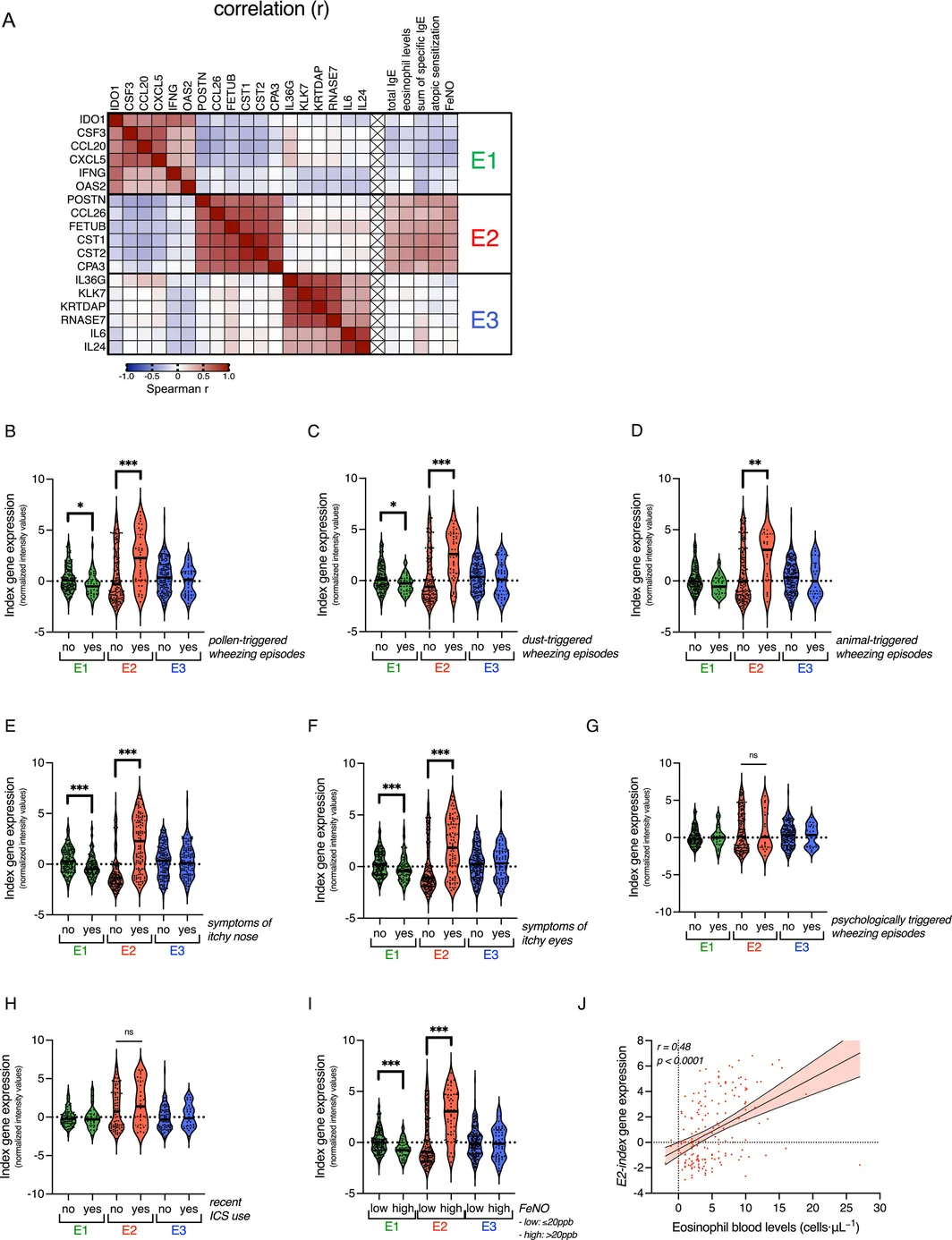
The E2 nasotype correlates with clinical symptoms and biomarkers of asthma in the ALLIANCE cohort. (A) Clinical parameters total serum IgE, blood eosinophil levels, the sum of specific IgE against allergens of the Euroimmune panel (sum_EuroImmun), atopy sensitization, and fractional exhaled nitric oxide (FeNO), were correlated with the E1, E2, and E3 index. The associated gene expression intensity is shown. Red boxes indicate high Spearman *r* values and blue boxes have low values. For visual guidance, genes are ordered by nasotype module (E1–E3); color intensity reflects within‐cohort Spearman correlation coefficients. (B) Violin plots depict gene expression abundance of E1 (green), E2 (red), and E3 (blue)‐nasotype index, calculated as mean of the E‐nasotype‐specific key markers in children with or without (B) wheezing episodes following exposure to pollen within the last 12 months (no: *N* = 136; yes: *N* = 39), (C) wheezing episodes following exposure to dust within the last 12 months (no: *N* = 132; yes: *N* = 43), (D) wheezing episodes following exposure to animals within the last 12 months (no: *N* = 152; yes: *N* = 23), (E) symptoms of itchy nose within the last 12 months (no: *N* = 145; yes: *N* = 111), (F) symptoms of itchy eyes within the last 12 months (no: *N* = 169; yes: *N* = 87), (G) psychologically triggered wheezing episodes within the last 12 months (no: *N* = 153; yes: *N* = 22), (H) recent ICS use (no: *N* = 83; yes: *N* = 26), (I) FeNO levels stratified into low: ≤ 20 ppb; intermediate/high: > 20 ppb (≤ 20 ppb: *N* = 169; > 20 ppb: *N* = 51). Two‐sided Mann–Whitney *U* tests compare the same E1, E2, and E3 indices of yes versus no parameters or intermediate/high versus low FeNO values. Statistical significances are indicated as *p < 0.05, **p < 0.01, ***p < 0.001. (J) Spearman correlation of eosinophil blood levels with *E2‐index* gene expression (*N* = 186). The graph depicts mean simple linear regression with a 95% confidence interval.

### 2.8 Low E1/High E2 and Asthma Persistence

Given the longitudinal design of the ALLIANCE cohort (NCT02496468), we assessed whether early epithelial immune states predict disease persistence. Children initially enrolled with preschool wheeze (age 1–6) were followed prospectively to their clinical outcome at age 6. Using matched nasal transcriptomes and metadata from individual participants, we compared nasotype expression at enrolment with subsequent asthma status. Children were classified into remission, residual symptoms, or probable asthma. Only those who progressed to asthma at age 6, but not children in remission, displayed a marked increase in E2‐nasotype expression after age 4, accompanied by downregulation of E1 (Figure 6A–C). Multivariate logistic regression adjusted for age, acute ENT symptoms, sensitization, and mutual influence of nasotypes confirmed this pattern (AUC = 0.90; Tables 3 and 4). The E1‐nasotype showed an inverse association with asthma persistence (OR = 0.15, 95% CI 0.03–0.81), whereas the E3‐nasotype showed a positive association with later disease progression (OR = 4.93, 95% CI 1.24–19.64) in the mutually adjusted model (Figure 6D,E). These findings indicate that loss of IFN‐γ‐linked epithelial programs (E1) and enhanced type‐2 polarization (E2) were associated with later persistent wheeze and asthma outcomes. Nasal epithelial immune states in early childhood may thus serve as molecular indicators of later asthma trajectories.

**FIGURE 6:**
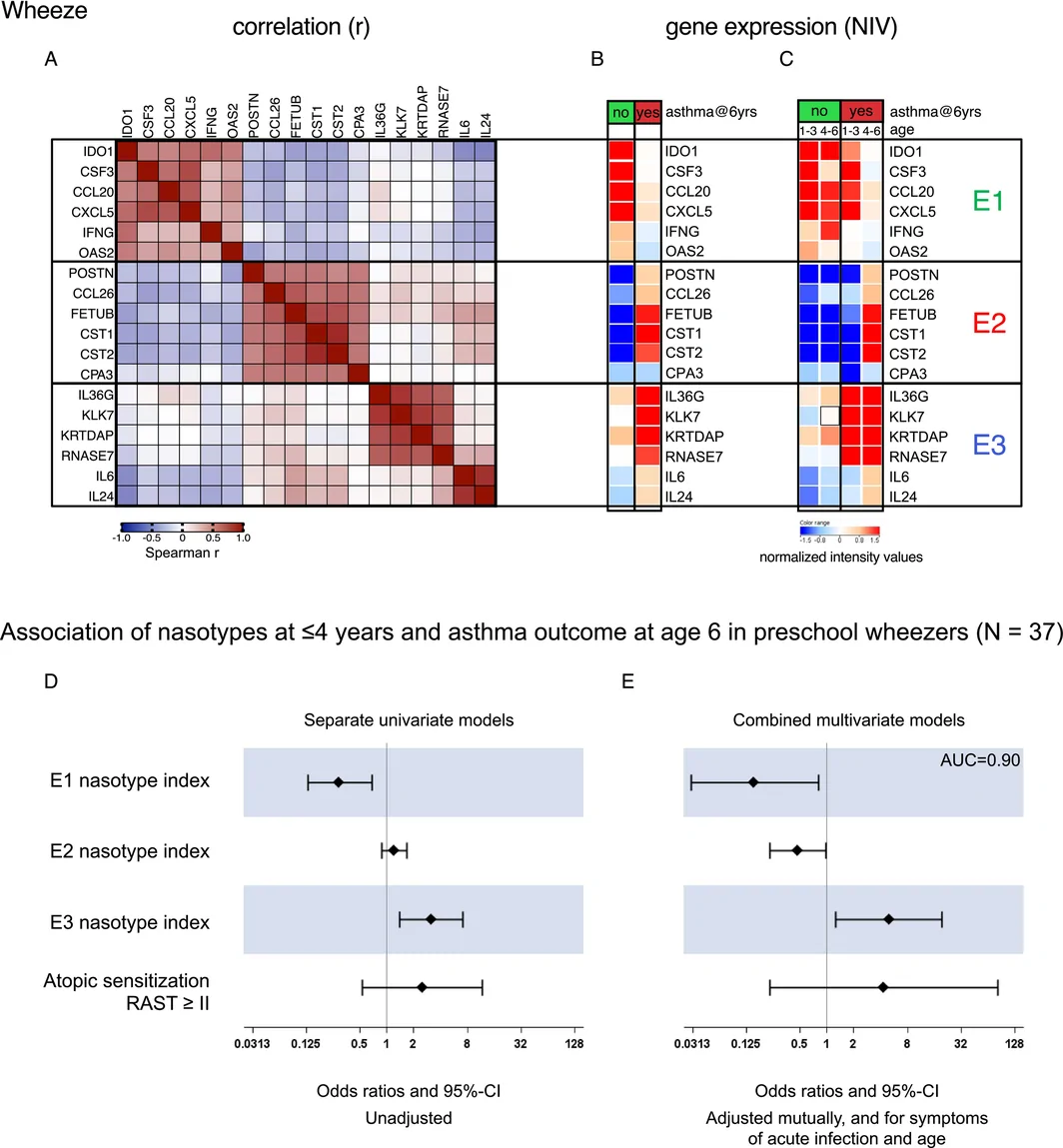
The E1 index is associated with symptom‐free outcomes by age 6 in preschool wheezers in the ALLIANCE cohort. Nasotype expression was assessed in children with preschool wheeze (age 1–6), and clinical outcomes were evaluated at age 6. (A) Heatmap of nasotype‐defining gene expression in nasal brushings; red indicates higher and blue lower relative expression. (B, C) Nasotype expression stratified by clinical outcome at age 6 (green = no asthma, red = asthma), shown for the full 1–6 age range (B) and stratified into 1–3 and 4–6 years (C). (D, E) Odds ratios from multivariable logistic regression models in preschool wheezers aged ≤ 4 years (*N* = 37) showing associations between nasotype indices and asthma persistence at age 6 years. Separate univariate models are shown in (D). Combined multivariable models including E1, E2, and E3 nasotype indices together with atopic sensitization (RAST ≥ II), adjusted for age and symptoms of acute ENT infection, are shown in (E). Odds ratios are displayed with 95% confidence intervals. Overall model discrimination is summarized by the area under the curve (AUC = 0.90).

**TABLE 3:** Clinical characteristics of preschool wheezers aged ≤ 4 years included in the predictive asthma outcome analyses.

			Total, *N* = 37		No asthma, *N* = 17		Asthma, *N* = 20	
Male gender		*N* (%)	37	24 (64.9%)	17	10 (58.8%)	20	14 (70.0%)
Age (years)		Mean (SD)	37	3.48 (0.93)	17	3.23 (1.13)	20	3.69 (0.67)
		Median [Q1, Q]		3.58 [3.00, 4.25]		3.33 [2.42, 3.92]		3.88 [3.21, 4.29]
Eosinophils (cells·μL^−1^)		Mean (SD)	37	352.28 (528.94)	17	481.90 (734.96)	20	242.10 (217.79)
		Median [Q1, Q4]		216.00 [99.00, 373.98]		248.00 [160.00, 390.00]		152.19 [84.00, 347.87]
Neutrophils (cells·μL^−1^)		Mean (SD)	37	3767.86 (1442.38)	17	3658.22 (1357.77)	20	3861.05 (1539.35)
		Median [Q1, Q4]		3578.25 [2818.56, 4346.10]		3636.30 [2730.00, 4346.10]		3577.73 [2860.40, 4367.39]
BMI (kg/m^2^)		Mean (SD)	37	16.12 (1.31)	17	15.97 (0.95)	20	16.24 (1.56)
		Median [Q1, Q4]		15.99 [15.27, 16.96]		16.00 [15.50, 16.50]		15.64 [15.10, 17.81]
Any exacerbation past 12 months		*N* (%)	37	20 (54.0%)	17	7 (41.2%)	20	13 (65.0%)
ICS use past 12 months		*N* (%)	37	23 (62.2%)	17	10 (58.8%)	20	13 (65.0%)
GINA	Controlled	*N* (%)	37	18 (48.6%)	17	8 (47.1%)	20	10 (50.0%)
	Partially controlled			14 (37.8%)		8 (47.1%)		6 (30.0%)
	Uncontrolled			5 (13.5%)		1 (5.9%)		4 (20.0%)
Parental hay fever		*N* (%)	37	20 (54.0%)	17	10 (58.8%)	20	10 (50.0%)
Parental eczema		*N* (%)	36	11 (30.6%)	16	3 (18.7%)	20	8 (40.0%)
Parental asthma		*N* (%)	37	12 (32.4%)	17	5 (29.4%)	20	7 (35.0%)
Atopic sensitization (RAST II)		*N* (%)	37	10 (27.0%)	17	3 (17.6%)	20	7 (35.0%)
ETS (≥ 10/day)		*N* (%)	36	2 (5.6%)	17	1 (5.9%)	19	1 (5.3%)
Maternal smoke exposure (pregnancy)		*N* (%)	37	3 (8.1%)	17	1 (5.9%)	20	2 (10.0%)

*Note:* Data are shown for preschool wheezers aged ≤ 4 years from the pediatric arm of the ALLIANCE cohort, stratified according to asthma outcome at age 6 years (“No asthma” versus “Asthma”).

**TABLE 4:** Association of nasotypes at ≤ 4 years and asthma outcome at age 6 in preschool wheezers (multivariable logistic regression models; *N* = 37).

≤ 4, *N* = 37									Additionally adjusted for acute ENT symptoms							
	Separate models unadjusted				Combined multivariate models adjusted for age				Separate models adjusted for acute ENT symptoms				Combined multivariate models adjusted for acute ENT symptoms and age			
	OR	95% CI	*p*	AUC	OR	95% CI	*p*	AUC	OR	95% CI	*p*	AUC	OR	95% CI	*p*	AUC
E1 index	0.29	0.13–0.68	**0.004**	0.81	0.18	0.05–0.73	**0.016**	0.90	0.25	0.09–0.68	**0.007**	0.80	0.15	0.03–0.81	**0.028**	0.90
E2 index	1.21	0.88–1.67	0.240	0.61	0.47	0.23–0.97	**0.041**		1.21	0.87–1.67	0.258	0.66	0.47	0.23–0.97	**0.040**	
E3 index	3.16	1.39–7.15	**0.006**	0.80	5.17	1.28–20.91	**0.021**		3.16	1.40–7.16	**0.006**	0.79	4.93	1.24–19.64	**0.024**	
Atopic sensitisation*	2.51	0.53–11.83	0.244	0.59	3.59	0.21–61.04	0.376		2.57	0.54–12.26	0.237	0.61	4.32	0.23–82.26	0.330	

*Note:* Significant *p*‐values are given in bold.

* RAST ≥ II.

## 3 Discussion

Here we identify three distinct mucosal transcriptional programs (E1, E2, and E3) triggered by type‐1, type‐2, and type‐3 immune responses. Throughout this study, E1–E3 are conceptualized as epithelial response programs reflecting mixed immune‐epithelial activation states rather than fixed epithelial states or immune cell‐defined endotypes. Asthmatic children displayed age‐associated differences in nasotype expression, reflecting distinct airway immune patterns across age groups. The three nasotypes were associated with prospectively assessed longitudinal outcomes: E1 was inversely associated with asthma persistence, E2 correlated with type‐2 inflammatory parameters and was selectively activated in wheezers who later developed asthma. In contrast, E3 was independently associated with disease persistence (AUC = 0.90).

Mechanistically, paracrine cytokine signaling from immune cells is sufficient to induce these epithelial states. We previously reported epithelial commitment to type‐1 and type‐2 states in vitro [11]. Building on our previous in vitro observations of type‐1 and type‐2 epithelial commitment, this study validates these findings in patients and extends these observations by defining an *IL‐36G*/*IL‐17*‐linked epithelial response program in nasal brush transcriptomes. Although shaped by canonical immune mediators, E1–E3 represent epithelial‐intrinsic states distinct from classical Th1/Th2/Th17 or M1/M2 polarization. Their characteristic mediators (*IDO1* for E1, *POSTN* and *CCL26* for E2, and *IL36G* and *RNASE7* for E3) show virtually no overlap within airway epithelial cells, underscoring their high program specificity.

Although epithelial cells expressing E1–E3 programs were identified in all epithelial subtypes, including basal, secretory, ciliated, and goblet cells, E1–E3 cells differ not only in immune signaling but also in structure and function. RNA velocity analysis suggested distinct transcriptional transitions of E2‐associated cells toward intermediate secretory subsets while retaining partial basal‐associated features compared with E1‐ and E3‐cells, although these dynamics were inferred from ALI cultures and may not fully reflect in vivo epithelial differentiation. These transcriptional differences coincide with distinct barrier phenotypes: E1 was associated with tight‐junction integrity (*ZO‐1, ZO‐3*), E3 with structural stabilization (*ZO‐2*, desmosomes, claudins), whereas E2 correlated with claudins linked to increased permeability (*CLDN2, 22, 24, 25*) but not with tight or adherens junctions. These findings agree with previous reports of barrier dysfunction in uncontrolled type‐2 inflammation [17]. Consistent with our observations, a recent study reported reduced expression of tight‐junction markers (*OCLN, TJP1*) in house‐dust‐mite allergy, consistent with barrier disruption during type‐2 inflammation [18]. Furthermore, the well‐described clinical shift from specific mucus components, *MUC5B* (healthy) to *MUC5AC* (asthma) [19, 20], parallels the E1‐to‐E2 transition, supporting a model in which impaired E1 signaling and unchecked E2 activation promote epithelial dysfunction and asthma development at school age.

Healthy children showed balanced mucosal immunity with proportionate nasotype expression, whereas young healthy children under age 6 and preschool wheezers predominantly expressed E1‐ and E3‐nasotypes. In contrast, children with wheeze from age 4 and school‐age asthmatics displayed a dominant E2‐nasotype correlated with atopic parameters. This shift reflects the classical type‐2 immunodominance underlying chronic T2‐asthma [9]. Although cross‐sectional by design, the contrasting E3 predominance in preschool wheeze and E2 predominance in school‐age asthma indicates age‐associated differences in mucosal immune states across development. Our multivariate models based on longitudinal metadata support nasotype‐associated disease persistence and highlight a potential early window for risk stratification and prevention. These findings reflect association‐based risk stratification.

The novel E3‐nasotype resembles type‐3 immunity and represents a pro‐inflammatory innate activation state potentially associated with immune maturation in early life. However, when combined with reduced E1 expression, this state was associated with later asthma persistence in our longitudinal outcome analysis. E1‐ and E3‐nasotypes correlated with antimicrobial peptides and TLRs, whereas E2 correlated negatively with *TLR7*, which recognizes single‐stranded RNA (ssRNA). E2‐dominance may therefore contribute to impaired viral recognition and antiviral defense; thus, to impaired antiviral defense against RNA viruses such as HRV and RSV, both primary triggers of asthma exacerbations.

Although *IFNG* belongs to E1, all other type‐I and type‐III interferons correlated strongly with the E3‐nasotype. This phenomenon, together with the antagonistic regulation of E1 versus E3, can explain the *GSDMB*‐related IFN‐swap previously observed with 17q21 risk variants [6] and may underlie the increased viral susceptibility in these children. The lack of physiological mucosal IFN expression in early life, recently identified as a key innate defense mechanism against viral respiratory infections [21], further supports this interpretation. The association of the pro‐inflammatory E3‐nasotype with infection symptoms and long‐term ICS requirement aligns with these findings.

Children with high E2 markers showed reduced NK and T‐cell markers but increased mast‐cell genes from age 9, consistent with a cellular skew toward mast‐cell‐dominated mucosal immunity. *IL33* correlated positively with E1 but negatively with E3, whereas *TSLP* correlated positively with E3 and negatively with E1. These interactions may reflect cytokine‐driven shifts from E1 to E2 (IFN‐γ‐induced IL‐33 [22]) or E3 to E2 (TSLP‐induced IL‐6 [23]) during allergic sensitization.

Replication analyses across the adult ALLIANCE arm and three external datasets (URECA, GRASS, CatEEC) supported the robustness of the E1 and E2 modules, whereas the E3 module showed weaker and less consistent within‐module correlation patterns across replication cohorts. Correlation with nasal phenotypes from U‐BIOPRED [16] further confirmed translational relevance: TAC1 genes (eosinophilic, FeNO‐high, POSTN‐high) aligned with E2, whereas TAC2 genes (neutrophilic, CRP‐high) corresponded to E1/E3. Although gene–gene correlations showed cohort‐specific heterogeneity, particularly for interferon‐ and IL‐17‐associated genes, the overall preservation of the modular E1/E2/E3 structure across three independent cohorts supports the biological relevance of the nasotype framework.

The identified E1–E3 nasotypes were derived using a hypothesis‐driven approach based on predefined cytokine axes and a focused set of epithelial immune‐related transcripts. Most analyses were based on cross‐sectional age‐group comparisons rather than longitudinal sampling, and observed differences may additionally reflect disease characteristics, sensitization, treatment exposure, infection status, or other cohort‐related factors. In addition, the ALI cytokine stimulation experiments represent controlled epithelial model systems that may not fully capture airway immune regulation in vivo.

In conclusion, the identification of three nasal epithelial immune states highlights distinct epithelial patterns associated with mucosal immune regulation in asthma. The age‐ and disease‐dependent activation of E1, E2, and E3 reflects key features of airway pathophysiology: E1 was associated with protection against asthma persistence, E2 drives barrier dysfunction and type‐2 inflammation, and E3 links innate activation to chronic asthma progression. These data establish nasal analytics as a minimally invasive approach to trace mucosal immune maturation and to define early epithelial trajectories that predispose to asthma persistence, defining critical windows for prevention and early intervention.

## 4 Methods

### 4.1 Study Population and Design

The clinical study design, the ethical approval, and the study protocol of the ALLIANCE cohort were previously published [24]. Details on study design, patient classification, clinical assessments, and follow‐up procedures are summarized in Table 1 (pediatric) and Table S27 (adult); participant flow, including exclusions and derivation of the final analysis dataset, is shown in Figure S1 [24].

Between 2013 and 2016, 265 genetically independent children and young adults with a median age of 10.0 years [1.0–20.0] were enrolled and regularly followed in the pediatric arm. Age‐related nasotype expression analyses were based on cross‐sectional comparisons between participants of different ages. Seventy‐nine healthy control children (median age: 12.0 [1.0–20.0]) without a history of asthma or preschool wheeze were enrolled, 81 children with at least two episodes of parent‐reported wheezing within the past 12 months classified as “preschool wheeze” (median age: 4.3 [1.1–7.9]), and 105 children with physician‐diagnosed asthma according to German and international GINA guidelines [25, 26] (median age: 13.1 [6.3–18.8]). Wheeze in children younger than six was not classified as asthma but considered a potential precursor phenotype.

Spirometry and fractional exhaled nitric oxide (FeNO) were assessed in all participants aged 6 years and older; spirometry data are expressed as age‐, height‐, and sex‐adjusted z‐score [https://gli‐calculator.ersnet.org]. FeNO measurements were also performed in children aged 4 to 5 years if they could perform the procedure reliably and if the measurement quality was acceptable. Laboratory tests included a differential blood count and specific IgE levels to 36 allergens (Euroline, Euroimmun). Atopic sensitization was defined as any specific IgE ≥ 0.7 kU/L. The sum of all specific IgE levels was calculated and log‐transformed to reflect the degree of sensitization. Exacerbations within the past 12 months were defined as either hospitalization for wheeze or the need for systemic corticosteroids. A questionnaire‐based disease activity score [9] was applied at the first follow‐up visit at or after 6 years of age to determine the persistence of disease among children initially enrolled as preschool wheezers. This score categorizes disease activity into asthma, asthma likely, residual symptoms, or remission. Further details regarding methods, study design, and definitions of clinical variables are provided in the Supporting Information and were previously published [24]. The adult arm comprised 25 healthy controls (median age: 61.0 years [26–79]) without prior asthma and 149 patients with physician‐diagnosed asthma (median age: 59.5 years [21–88]) [9].

### 4.2 Statistical Analysis

Differential gene expression analyses were performed using moderated *t*‐tests with Benjamini‐Hochberg correction for multiple testing. Group comparisons were assessed using two‐sided Mann–Whitney *U* tests, and correlations were analyzed using Spearman's rank correlation coefficients. Associations between nasotype indices, clinical parameters, and asthma outcomes were evaluated using multivariate linear and logistic regression models adjusted for relevant covariates, including age and sensitization status, as indicated. Single‐cell RNA‐seq analyses were performed using Scanpy and Seurat frameworks. Statistical analyses were performed in R (v4.4.0), SAS (v9.4), and GraphPad Prism (v10.4.1), unless otherwise stated. Further methodological and statistical details are provided in the Supporting Information.

## Author Contributions

Conceptualization: C.A.J., C.B.S.‐W Methodology: C.A.J., H.C., M.O., A.A.G., U.M.Z., A.E., M.F., F.D., M.B., Benjamin Schubert, U.P. Investigation and sample processing: C.A.J., H.C., M.O., A.A.G., U.M.Z., A.E., M.F., M.W., A.M.C. Clinical cohort management and metadata curation: S.I., N.M., J.O., M.W., R.G., A.‐M.D., S.F., C.S., T.T., S.K.‐R., G.H., T.B., K.F.R., F.B., M.V.K., Bianca Schaub, E.M. Transcriptomic and bioinformatic analyses: C.A.J., A.A.G., F.D., M.B., Benjamin Schubert. Single‐cell sequencing and in vitro epithelial experiments: C.A.J., H.C., M.O., A.E., M.F., U.P. Statistical analysis: C.A.J., S.I., H.C., A.A.G., F.D., Benjamin Schubert. Predictive model development, recalculation, and interpretation: S.I. Visualization: C.A.J., H.C., A.A.G., F.D. Clinical and biological interpretation: C.A.J., S.I., N.M., M.W., R.G., A.‐M.D., S.F., C.S., T.T., S.K.‐R., G.H., T.B., K.F.R., F.B., M.V.K., A.M.C., Bianca Schaub, E.M., C.B.S.‐W. Technical supervision and coordination of sample processing: U.M.Z. Training of medical staff in nasal sampling: A.M.C. Supervision: C.A.J., C.B.S.‐W., Bianca Schaub, E.M., G.H., K.F.R., T.B., M.V.K., U.P. Project administration: C.A.J., U.M.Z., C.B.S.‐W. Funding acquisition: C.B.S.‐W., Bianca Schaub, E.M., G.H., K.F.R., U.P., A.M.C. Writing – original draft: C.A.J. Writing – review and editing: all authors. All authors approved the final version of the manuscript and agree to be accountable for all aspects of the work.

## Funding

This study was developed by the German Center for Lung Research (DZL), funded by the Federal Ministry of Education and Research (BMBF), the Bavarian State Office for Health and Food Safety (LGL; K1‐2497‐GLB‐20‐V4) of the “Gesund.Leben.Bayern.” initiative of the Bavarian State Ministry of Health and Care, by grants from Deutsche Forschungsgemeinschaft RTG2668 (Project A5, Project‐ID: 435874434) to C.B. Schmidt‐Weber and by grants from Deutsche Forschungsgemeinschaft RTG2668 (Project A1, Project‐ID: 435874434) to C.A. Jakwerth. C.S. is supported by the Universities of Giessen and Marburg Lung Center (UGMLC), University Hospital Giessen and Marburg (UKGM) research funding according to article 2, section 3 cooperation agreement, and the Deutsche Forschungsgemeinschaft (DFG, German Research Foundation)‐SFB 1021 (Project Number 197785619), KFO 309 (Project Number 284237345), and SK 317/1‐1 (Project Number 428518790) as well as by the Foundation for Pathobiochemistry and Molecular Diagnostics outside the submitted work. B.S. was supported by Federal Ministry of Education and Research (Bundesministerium für Bildung und Forschung, BMBF) as part of the DZL, German Lung Centre, DZL2024‐27, 82DZL033C2 and German Center for Child and Adolescent Health (DZKJ); German Center for Child and Adolescent Health (DZKJ), SCHA SCHA‐997/9‐1, SCHA‐997/10‐1, SCHA 997/11‐1. F.D. is supported by the Helmholtz Association under the joint research school “Munich School for Data Science—MUDS” and the BMBF grant DeepTCR (project 031L0290A). F.D. acknowledges financial support from the Joachim Herz Stiftung.

## Conflicts of Interest

C.A.J. reports grants from Federal Ministry of Education and Research (BMBF) for the German Center for Lung Research (DZL), grants from German Research Foundation (DFG Grant No. 398577603), grants from Bavarian State Office for Health and Food Safety (K1‐2497‐GLB‐20‐V4) during the conduct of the study; grants from Federal German Ministry of Education and Research (BMBF ABROGATE), Max Zeller Söhne AG, Else‐Kröner‐Fresinius‐Stiftung, DFG Exzellenzinitiative TUM International Graduate School of Science and Engineering (IGSSE) (JADS‐Project: PANORAMA), and from DFG Graduiertenkolleg RTG2668 (Project A1, Project‐ID: 435874434) outside the submitted work. M.W. reports grants from Federal Ministry of Research and Education, during the conduct of the study; grants from Novartis, personal fees from Chiesi, grants from Muko e.V., outside the submitted work. U.M.Z. reports grants from Federal Ministry of Education and Research (BMBF) for the German Center for Lung Research (DZL), grants and personal fees from German Research Foundation (DFG Grant No. 398577603), grants and personal fees from Bavarian State Office for Health and Food Safety (K1‐2497‐GLB‐20‐V4), during the conduct of the study; grants from European Institute of Technology (EIT), grants from CFi‐aktiv e.V. Mukoviszidose Selbsthilfe Südbayern outside the submitted work. R.G. reports grants from Federal Ministry of Education and Research (BMBF) for the German Center for Lung Research (DZL). A.‐M.D. reports grants from BMBF/DLR: German center for Lung Research, during the conduct of the study; grants from BMBF/DLR: CALM QE, outside the submitted work; funding to institution for clinical study conduct by Vertex Pharmaceuticals Inc. and OM Pharma, personal remuneration for advisory board opinions (Vertex Pharmaceuticals Inc.), medical expert advice (Austrian Society for Rare Diseases, GlaxoSmithKline, Oxford University), teaching Kinder‐ und Jugendpsychotherapie‐Ambulanz Universität Marburg, medical writing (Hogrefe Publishing, Vertex Pharmaceutical Inc., Christiane‐Herzog‐Stiftung), unpaid leadership role in clinical trial networks and advocacy groups (ECFS CTN, c4c, Muko e.V.), outside of this publication. C.S. reports research grants by the Universities Giessen and Marburg Lung Center (UGMLC), the German Center for Lung Research (DZL), University Hospital Giessen and Marburg (UKGM) research funding according to article 2, section 3 cooperation agreement, and the Deutsche Forschungsgemeinschaft (DFG, German Research Foundation)‐SFB 1021 (Project Number 197785619), KFO 309 (Project Number 284237345), and SK 317/1‐1 (Project Number 428518790) and by the Foundation for Pathobiochemistry and Molecular Diagnostics, and consultancy and research funding by Bencard Allergie and Thermo Fisher Scientific. S.F. reports research funding (BMBF). G.H. reports grants from the BMBF for the Deutsches Zentrum für Lungenforschung (DZL), during the conduct of the study; from the Deutsche Forschungsgemeinschaft (DFG, German Research Foundation) under Germany's Excellence Strategy—EXC 2155 “RESIST”—Project ID 39087428, German Federal Ministry of Education and Research (BMBF), CHAMP (01GL1742F), Niedersachsen Vorab Foundation (INDIRA), and the Else Kröner‐Fresenius Foundation (EKFS) for the Research College TITUS, the foundation Niedersachsen Vorab for the research project INDIRA, consulting fees from Sanofi GmbH, lecture fees from MedUpdate, Abbvie, outside the submitted work. T.B. reports grants from BMBF: Unrestricted research grant for the German Center for Lung Research (DZL), during the conduct of the study; personal fees from AstraZeneca, personal fees from GlaxoSmithKline, personal fees from Novartis, personal fees from Roche, personal fees from Chiesi, personal fees from Boeringer Ingelheim, personal fees from Pfizer, personal fees from MSD outside the submitted work. K.F.R. is co‐director of the DZL and reports grants from the BMBF for the Deutsches Zentrum für Lungenforschung (DZL), during the conduct of the study; other grants from Astra Zeneca, Berlin Chemie, Boehringer Ingelheim, Chiesi, and Sanofi Regeneron, outside the submitted work. He is co‐founder of rnatics. M.V.K. reports grants from the BMBF for the Deutsches Zentrum für Lungenforschung (DZL), during the conduct of the study; others from Sanofi Aventis GmbH, others from Allergopharma GmbH, others from Chiesi GmbH, personal fees from Infectopharm GmbH, personal fees from Nutricia GmbH, outside the submitted work. A.M.C. reports grants from German Center for Lung Research (DZL) during the conduct of the study; grants, speaker honoraria, consultancy or advisory fees and/or research support and other, all via Technical University of Munich from ALK‐Abello, AstraZeneca, Bencard/Allergen Therapeutics, ASIT Biotech, GSK, Hippo Dx, Novartis, LETI, Roche, Sanofi, Regeneron, Zeller, grants from Federal German Ministry of Education and Research, grants from European Institute of Technology, all outside this submitted work. B. Schaub grants from Federal German Ministry of Education and Research (BMBF), grants from DFG, during the conduct of the study; grants from DFG, from Federal German Ministry of Education and Research (BMBF), other from GSK, Sanofi, Novartis, outside the submitted work. E.M. reports grants from German Federal Ministry of Education and Research (BMBF), German Center for Lung Research (DZL), during the conduct of the study; grants from Bavarian State Ministry of Health and Care for “URS Study,” 01/21, grants from “Impact Chip Study,” OM Pharma S.A., grants from Go Bio Initial Grant, BMBF (Federal Ministry of Education and Research), grants from European Research Council award, grants from “BEAR Study,” O.M. Pharma S.A., personal fees from Chinese University of Hongkong, European Commission, HiPP GmbH & Co KG, AstraZeneca, Imperial College London, OM Pharma, ALK‐Abello Arzneimittel GmbH, personal fees from Massachusetts Medical Society, Springer‐Verlag GmbH, Elsevier Ltd., Böhringer Ingelheim International GmbH, European Respiratory Society (ERS), Universiteit Utrecht, Faculteit Diergeneeskunde, Universität Salzburg, Springer Medizin Verlag GmbH, Japanese Society of Pediatric Allergy and Clinical Immunology (JSPACI), Klinikum Rechts der Isar, University of Colorado, Paul‐Martini‐Stiftung, Astra Zeneca, Imperial College London, Children's Hospital Research Institute of Manitoba, Kompetenzzentrum für Ernährung (Kern), OM Pharma S.A., Swedish Pediatric Society for Allergy and Lung Medicine, Chinese College of Allergy and Asthma (CCAA), ALK‐Abello Arzneimittel GmbH, Abbott Laboratories, Deutscher Apotheker Verlag GmbH & Co. KG, Japanese Society of Allergology, British Society for Asthma and Clinical Immunology, American Academy of Allergy, Asthma & Immunology, personal fees from Verein zur Förderung der Pneumologie am Krankenhaus Großhansdorf e.V., Pneumologie Developpement, Mondial Congress & Events GmbH & Co. KG, American Academy of Allergy, Asthma & Immunology, Imperial College London, Margaux Orange, Volkswagen Stiftung, Böhringer Ingelheim International GmbH, European Respiratory Society (ERS), Universiteit Utrecht, Faculteit Diergeneeskunde, Österreichische Gesellschaft f. Allergologie u. Immunologie, Massachusetts Medical Society, OM Pharma S. A., Hanson Wade Ltd., iKOMM GmbH, DSI Dansk Borneastma Center, American Thoracic Society, HiPP GmbH & Co KG, Universiteit Utrecht, Faculteit Bètawetenschappen, ALK‐Abello Arzneimittel GmbH, Deutsches Zentrum für Lungenforschung (DZL), Fabio Luigi Massimo Ricciardolo/Contatto S.r.l., Fraunhofer ITEM Hannover, MCCA Institut für Immunologie Uni Wien, Swiss Institute of Allergy and Asthma Research (SIAF) Davos (Associated Institute of the University of Zurich), MHH (Medizinische Hochschule Hannover), European Respiratory Socierty, Natasha Allergy Research Foundation, Deutsche Forschungsgemeinschaft, Gordon Research Conferences, Socieded Chilena de Enfermedades Respiratorias, Arla, Universität Leiden, American Academy of Allergy, Asthma & Clinical Immunology, Deutsche Forschungsgemeinschaft (DFG), ERS, personal fees from Member of the EXPANSE (funded by European Commission) Scientific Advisory Board, Member of the BEAMS External Scientific Advisory Board (ESAB), Member of the Editorial Board of “The Journal of Allergy and Clinical Immunology: In Practice,” Member of the Scientific Advisory Board of the Children's Respiratory and Environmental Workgroup (CREW), Member of the International Scientific & Societal Advisory Board (ISSAB) of Utrecht Life Sciences (ULS), University of Utrecht, Member of External Review Panel of the Faculty of Veterinary Science, University of Utrecht, Member of the Selection Committee for the Gottfried Wilhelm Leibniz Programme (DFG), Member of the International Advisory Board of Asthma UK Centre for Applied Research (AUKCAR), Member of the International Advisory Board of “The Lancet Respiratory Medicine,” Member of the Scientific Advisory Board of the CHILD (Canadian Healthy Infant Longitudinal Development) study, McMaster University, Hamilton, Canada, Asthma UK Centre for Applied Research, Pediatric Scientific Advisory Board Iceland, Abbott Allergy Risk Reduction Advisory Board, outside the submitted work; In addition, Prof. Dr. Dr. von Mutius has a patent No. PCT/EP2019/085016 (Barn dust extract for the prevention and treatment of diseases) pending, royalties paid to ProtectImmun for patent EP2361632 (Specific environmental bacteria for the protection from and/or the treatment of allergic, chronic inflammatory and/or autoimmune disorders, granted on 19 March 2014), and patents EP1411977 (Composition containing bacterial antigens used for the prophylaxis and the treatment of allergic diseases, granted on 18 April 2007), EP1637147 (Stable dust extract for allergy protection, granted on 10 December 2008), and EP 1964570 (Pharmaceutical compound to protect against allergies and inflammatory diseases, granted on 21 November 2012) licensed to ProtectImmun. Patent EP21189353.2.2021. von Mutius E, Rankl B, Bracher F, Müller C, Walker A, Hauck SM, Merl‐Pham J, inventors; PROTEINS IDENTIFIED FROM BARN DUST EXTRACT FOR THE PREVENTION AND TREATMENT OF DISEASES. Patent PCT/US2021/016918.2021. Martinez FD, Vercelli D, Snyder SA, von Mutius E, Pivniouk V, Marques dos Santos M, inventors; THERAPEUTIC FRACTIONS AND PROTEINS FROM ASTHMA‐PROTECTIVE FARM DUST. Patent EP21189353.2.2021. von Mutius E, Rankl B, Bracher F, Müller C, Walker A, Hauck SM, Merl‐Pham J, Adler H, Yildirim A.Ö., Sattler M, Santos Dias Mourao A, Borggräfe J, O'Connor P.D., Plettenburg O, inventors; PROTEINS IDENTIFIED FROM BARN DUST EXTRACT FOR THE PREVENTION AND TREATMENT OF DISEASES pending, and a patent Elsevier GmbH, Georg Thieme Verlag, Springer‐Verlag GmbH, Elsevier Ltd., Springer Nature Group with royalties paid. C.B.S.‐W. reports grants from Federal Ministry of Research and Education, during the conduct of the study. He received personal fees from Sanofi, Leti and grants from Zeller and Allergopharma, outside the submitted work. S.K.‐R. reports membership of the Scientific Advisory Board “Establishing Natural History in an Advanced New CF Care Era” (ENHANCE) Study, “Real World Clinical Outcomes with Novel Modulator Combinations in People with CF” (RECOVER) Study, HTA Project: NIHR131889 UK (“A randomised open label trial to assess change in respiratory function for people with CF established on triple combination therapy (Kaftrio) after rationalisation of nebulised mucoactive therapies”) (the CF‐STORM trial), and Scientific Advisory Board for BiomX and PureIMS outside the submitted work. F.B. reports grants from the BMBF for the Deutsches Zentrum für Lungenforschung (DZL) as well as grants from the BMBF for post infection research (post COVID). F.D. is supported by the Helmholtz Association under the joint research school “Munich School for Data Science—MUDS” and acknowledges financial support from the Joachim Herz Stiftung. B. Schubert reports grants from German Federal Ministry of Education and Research (BMBF), German Center for Lung Research (DZL), Deutsche Forschungsgemeinschaft (DFG), and the Helmholtz Association during the conduct of the study. H.C. reports support from Deutsche Forschungsgemeinschaft (RTG 2668 (Project A5, Project‐ID: 435874434) to Prof. Dr. Schmidt‐Weber). U.P. reports grants from Bavarian Ministry of Science and Arts, grants from European Union's Horizon 2020 research and innovation programme, grants from Helmholtz Association's Initiative and Networking Fund, during the conduct of the study; grants from German Research Foundation (DFG), personal fees from GILEAD, personal fees from Sanofi, personal fees from Aligos, nonfinancial support from Roche Diagnostics, outside the submitted work S.I., M.O., A.E., A.A.G., M.F., M.B., N.M., J.O., T.T. have nothing to disclose.

## Supporting information


**Figure S1:** Study populations included in the discovery and replication analyses. Flowchart of sample selection in the pediatric and adult ALLIANCE cohorts. The source cohort comprised 561 pediatric and 224 adult samples; 18 siblings were excluded from the pediatric arm. Nasal transcriptome analyses included 265 genetically independent children and 174 adults. Independent replication cohorts comprised URECA (*N* = 353), GRASS (59 participants; 256 samples), and CatEEC (19 participants; 256 samples).
**Figure S2:** Validation of E1‐E3 expression programs in adults with asthma from the ALLIANCE cohort. Spearman correlation matrices of the six highest‐ranked E1‐, E2‐, and E3‐associated genes in all genetically independent adults with asthma (A; *N* = 149) and in participants aged 18–39 years (B; *N* = 19), 40–64 years (C; *N* = 80), and ≥ 65 years (D; *N* = 50). Blue and red indicate negative and positive correlations, respectively. (E) Normalized gene‐expression intensity in adults with asthma stratified by age group. The color scale ranges from −1.5 to 1.5 normalized intensity values. Asterisks indicate differential expression between participants aged ≥ 65 and 18–39 years (moderated *t*‐test, *p* < 0.05 and fold change ≥ 1.5).
**Figure S3:** Replication of E1‐E3 gene‐expression programs in independent nasal transcriptome cohorts. Spearman correlation matrices of E1‐, E2‐, and E3‐associated genes in URECA (A; *N* = 353; one sample per participant; GSE145505), GRASS (B; 256 samples from 59 participants; GSE206151), and CatEEC (C; 256 samples from 19 participants; GSE129748). The color scale ranges from Spearman's *ρ* = −1 to *ρ* = 1.
**Figure S4:** E1–E3 expression and epithelial morphology in differentiated airway epithelial cultures. (A) Expression of E1‐, E2‐, and E3‐associated genes in the corresponding single‐cell E states, shown as violin plots of transcript copy numbers. (B) Representative hematoxylin and eosin and periodic acid‐Schiff staining of differentiated NHBE air‐liquid interface cultures maintained with or without IL‐4. Images were acquired at 40× magnification and represent three independent cultures.
**Figure S5:** Developmental trajectories of E1, E2, and E3 epithelial states. Distribution of E1, E2, and E3 cells across epithelial cell types under all treatment conditions (A) and after IL‐4 treatment (B). (C) Cell‐type composition within each E state. (D) UMAP representation of NHBE cells differentiated for 12 days at air–liquid interface under medium or IL‐4 conditions (50 ng/mL, added basolaterally every 48 h). Basal, ciliated, club, secretory, pulmonary neuroendocrine, tuft, and ionocyte populations are indicated. Palantir pseudotime trajectories are shown for all cells (E), E1 cells (G), E2 cells (H), and E3 cells (I), with corresponding coarse‐grained transition matrices in F and J–L. Colors indicate progression from early to late pseudotime. Pseudotime distributions are shown by E state (M) and treatment condition (N).
**Figure S6:** Cell‐fate probabilities and pathway enrichment across E1–E3 epithelial trajectories. Violin plots show cell‐fate probabilities of basal E1 (A), E2 (B), and E3 (C) cells for differentiation toward basal, club, four secretory, and ciliated cell states. Initial states (D–F) and terminal states (G–I) are shown for the corresponding E1‐E3 trajectories. Gene‐set enrichment analyses compare secretory E1 versus E2 cells (J, K) and basal E1 or E3 versus E2 cells (L, M).
**Figure S7:** Age‐resolved leukocyte‐marker expression across epithelial nasotypes and disease groups. (A) Leukocyte cell‐type marker expression in 265 genetically independent children with high E1, E2, or E3 index expression, defined as quintile 5, and stratified into age groups of 1–3, 4–6, 7–9, 10–12, 13–15, and 16–18 years. (B) Corresponding age‐resolved expression in healthy children and children with wheeze or asthma. Heatmaps show normalized intensity values ranging from −1.5 to 1.5.
**Figure S8:** Correlations between epithelial nasotypes and U‐BIOPRED transcript‐associated clusters. Spearman correlation matrix of the six highest‐ranked E1‐, E2‐, and E3‐associated genes and U‐BIOPRED transcript‐associated clusters. (A) Correlation coefficients, with blue and red indicating negative and positive correlations, respectively. (B) Corresponding *p* values, with shading ranging from light gray for *p* < 0.05 to dark gray for *p* < 0.0001.

**Table S1‐1:** Correlation matrix of nasotypes from all pediatric samples (*N* = 265)—Spearman *r* values.
**Table S1‐2:** Correlation matrix of nasotypes from all pediatric samples (*N* = 265)—Spearman *p* values.
**Table S2:** Seed correlation ranking and within‐module correlations of final nasotype marker genes.
**Table S3:** Ordered trends of predefined E1‐, E2‐, and E3‐associated genes across their respective cytokine seed‐variable quintiles.
**Table S4‐1:** Correlation matrix of nasotypes from all adult samples of the ALLIANCE cohort (*N* = 149)—Spearman *r* values.
**Table S4‐2:** Correlation matrix of nasotypes from adult samples of age group 18–39 (*N* = 19)—Spearman *r* values.
**Table S4‐3:** Correlation matrix of nasotypes from adult samples of age group 40–64 (*N* = 88)—Spearman *r* values.
**Table S4‐4:** Correlation matrix of nasotypes from adult samples of age group ≥ 65 (*N* = 50)—Spearman *r* values.
**Table S5:** All three nasotypes: Significant gene expression changes (*p* < 0.05, FC > 1.5) comparing adult patients with asthma aged ≥ 65 years versus adult patients with asthma aged 18–39 years.
**Table S6:** Correlation matrix of nasotypes from all pediatric nasal samples from the URECA cohort (*N* = 353)—Spearman *r* values.
**Table S7:** Correlation matrix of nasotypes from all nasal samples from the GRASS cohort (*N* = 256)—Spearman *r* values.
**Table S8:** Correlation matrix of nasotypes from all nasal samples from the CatEEC cohort (*N* = 256)—Spearman *r* values.
**Table S9:** Trajectory analysis—Driver genes of all different cellular subgroups (terminal states) (Figure 2).
**Table S10:** Trajectory analysis—Driver genes of different E1 cellular subgroups (terminal states) (Figure 2).
**Table S11:** Trajectory analysis—Driver genes of different E2 cellular subgroups (terminal states) (Figure 2).
**Table S12:** Trajectory analysis—Driver genes of different E3 cellular subgroups (terminal states) (Figure 2).
**Table S13:** Significant gene expression changes based on scRNASeq comparing secretory cells of different E‐types (Figure 2).
**Table S14‐1:** Cluster‐to‐cell type annotation mapping of cytokine‐stimulated NHBE ALI scRNA‐seq cultures (Figure 3).
**Table S14‐2:** Significant marker genes across epithelial scRNA‐seq clusters (Figure 3).
**Table S15:** Relative E‐state fractions across cytokine stimulation conditions in NHBE ALI cultures (Figure 3).
**Table S16:** Donor‐centered E‐state expression scores across cytokine stimulation conditions (Figure 3).
**Table S17:** Correlation matrix of nasotypes from healthy pediatric samples (*N* = 79)—Spearman *r* values.
**Table S18:** Correlation matrix of nasotypes from pre‐school wheeze pediatric samples (*N* = 81)—Spearman *r* values.
**Table S19‐1:** Significant differential expression of E1/E2/E3 nasotype‐associated genes in children with preschool wheeze versus healthy (Benjamini‐Hochberg adjusted *p* < 0.05, fold change > 1.5).
**Table S19‐2:** Significant differential expression of E1/E2/E3 nasotype‐associated genes in children with asthma versus wheeze (Benjamini‐Hochberg adjusted *p* < 0.05, fold change > 1.5).
**Table S20‐1:** Significant differential expression of E1/E2/E3 nasotype‐associated genes in children with asthma versus healthy (Benjamini‐Hochberg adjusted *p* < 0.05, fold change > 1.5).
**Table S20‐2:** Significant differential expression of E1/E2/E3 nasotype‐associated genes in children with asthma versus preschool wheeze (Benjamini‐Hochberg adjusted *p* < 0.05, fold change > 1.5).
**Table S21:** Correlation matrix of nasotypes from all pediatric samples (*N* = 265) with TAC phenotypes (U‐BIOPRED study)—Spearman *r* values.
**Table S22:** Association of age and gene expression of the E1, E2, and E3 index genes: beta coefficient and standard error (SE) based on linear regression.
**Table S23:** Association of disease status and gene expression of the E1, E2, and E3 index genes: beta coefficient and standard error (SE) based on linear regression.
**Table S24:** Significant differential expression of E1/E2/E3 nasotype‐associated genes in healthy children aged 1–6 years versus 13–18 years (Benjamini‐Hochberg adjusted *p* < 0.05, fold change > 1.5).
**Table S25:** Significant differential expression of E1/E2/E3 nasotype‐associated genes in preschool wheezers aged 4–6 years versus 1–3 years (Benjamini‐Hochberg adjusted *p* < 0.05, fold change > 1.5).
**Table S26‐1:** Correlation matrix of nasotypes from children with wheeze or asthma with atopic parameters—Spearman *r* values.
**Table S26‐2:** Correlation matrix of nasotypes from children with wheeze or asthma with atopic parameters—Spearman *p* values.
**Table S27:** Subject characteristics of healthy and asthmatic adults of the ALLIANCE cohort (DZL).
**Table S28:** Association of population characteristics and gene expression of the E1 index gene: beta coefficient and standard error (SE) based on linear regression.
**Table S29:** Association of population characteristics and gene expression of the E2 index gene: beta coefficient and standard error (SE) based on linear regression.
**Table S30:** Association of population characteristics and gene expression of the E3 index gene: beta coefficient and standard error (SE) based on linear regression.
**Table S31:** Candidate list of epithelial secreted/immune‐related transcripts set.

**Data S1:** Supplementary methods.

## Data Availability

The single‐cell RNA‐seq data generated in this study are available at the Gene Expression Omnibus under accession number GSE203248. Additional datasets generated in this study will be publicly available upon publication. The nasal bulk transcriptomic data are deposited under accession GSE252637. All other data supporting the findings of this study are available within the article and its Supporting Information files, or from the corresponding author upon reasonable request.
