# Supplementary material for: Nasal Epithelial Immune Signatures Are Associated With Age‐Dependent Asthma Trajectories in Early Life
Source: Allergy. 2026 Sep 6;81(10):3533–53. doi: 10.1111/all.70477 (PMC13646873; doi:10.1111/all.70477)

Figure S1

A

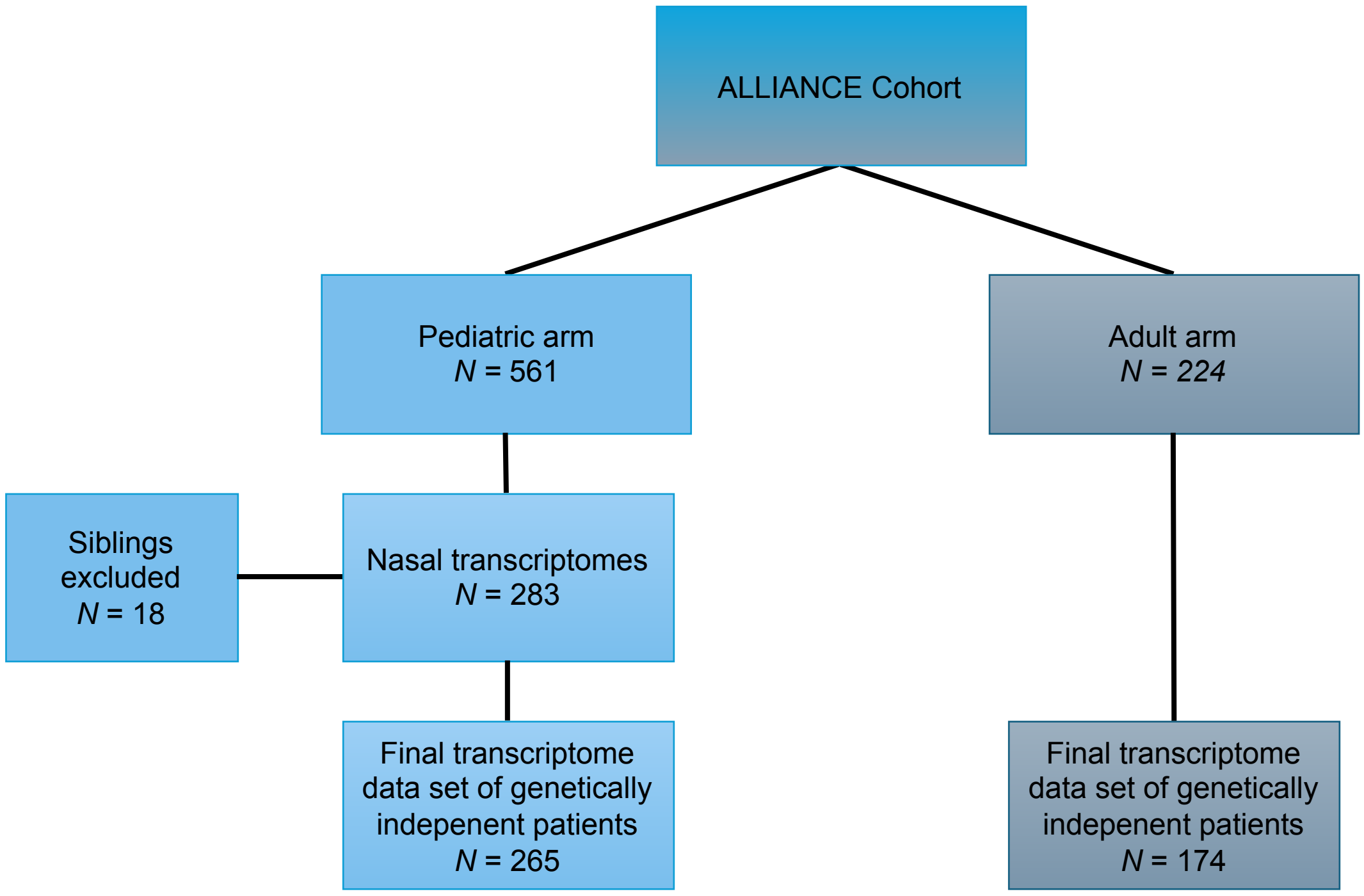

ALLIANCE Cohort
Pediatric arm
N = 561
Adult arm
N = 224
Siblings excluded
N = 18
Nasal transcriptomes
N = 283
Final transcriptome data set of genetically indepenent patients
N = 265
Final transcriptome data set of genetically indepenent patients
N = 174

B

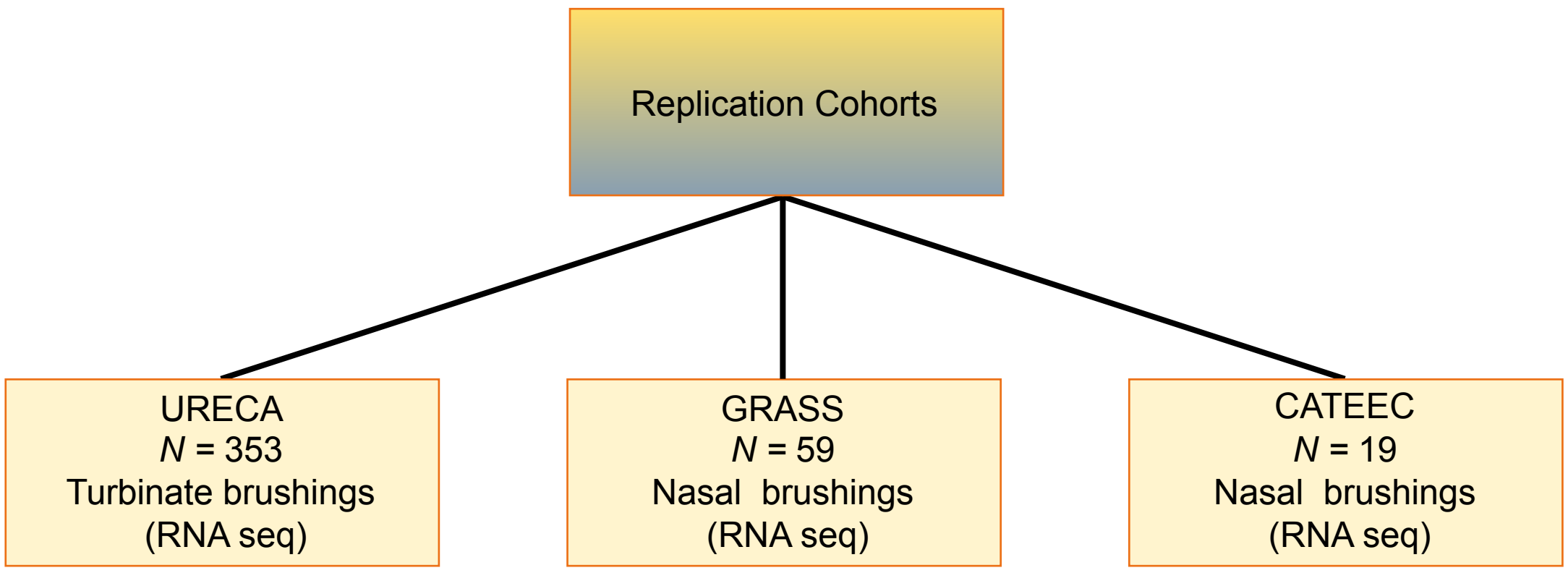

Replication Cohorts
URECA
N = 353
Turbinate brushings
(RNA seq)
GRASS
N = 59
Nasal brushings
(RNA seq)
CATEEC
N = 19
Nasal brushings
(RNA seq)

Figure S2

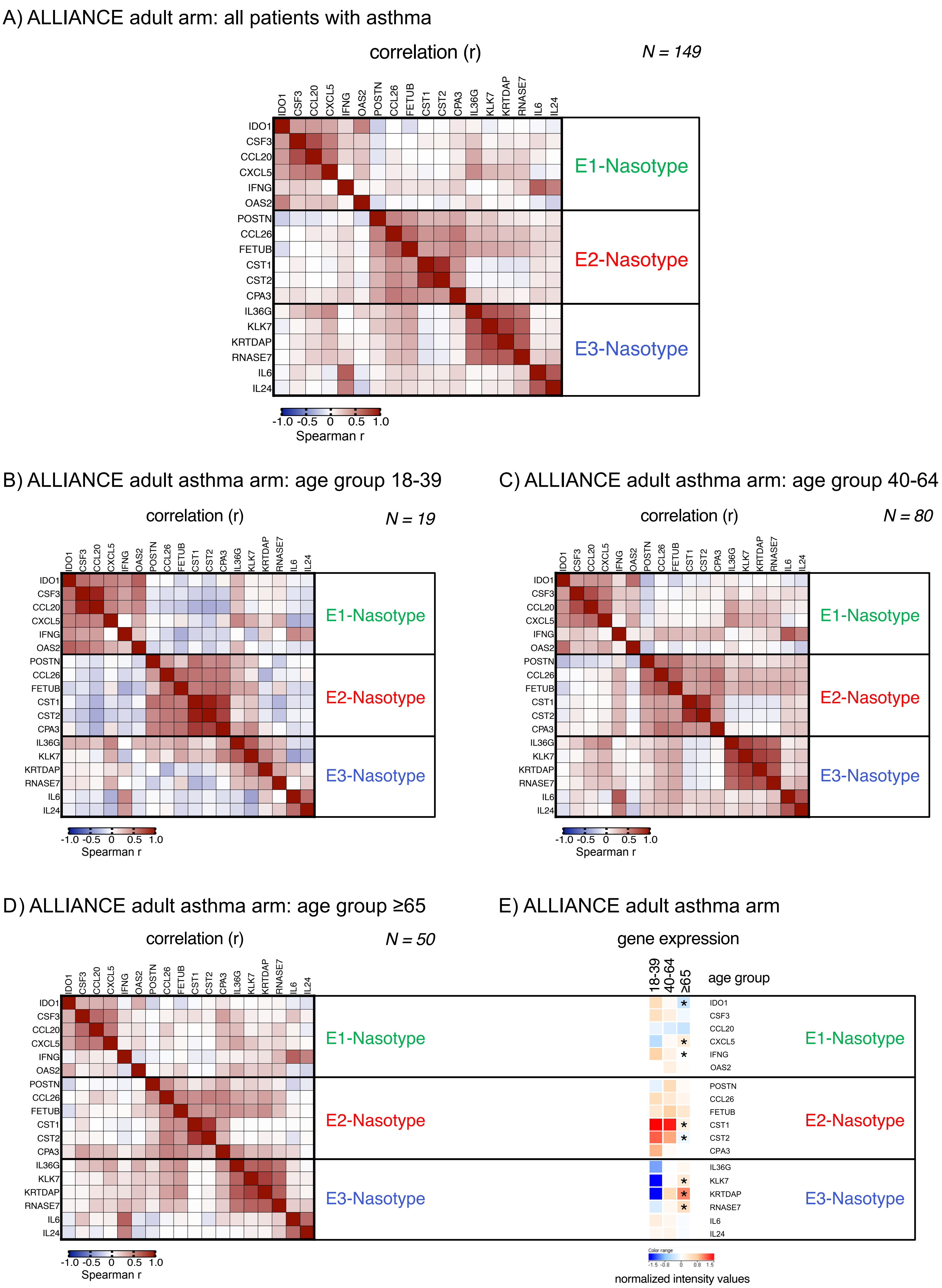

A) ALLIANCE adult arm: all patients with asthma
correlation (r)
N = 149
IDO1
CSF3
CCL20
CXCL5
IFNG
OAS2
POSTN
CCL26
FETUB
CST1
CST2
CPA3
IL36G
KLK7
KRTDAP
RNASE7
IL6
IL24
E1-Nasotype
E2-Nasotype
E3-Nasotype
-1.0 -0.5 0 0.5 1.0
Spearman r
B) ALLIANCE adult asthma arm: age group 18-39
correlation (r)
N = 19
E1-Nasotype
E2-Nasotype
E3-Nasotype
-1.0 -0.5 0 0.5 1.0
Spearman r
C) ALLIANCE adult asthma arm: age group 40-64
correlation (r)
N = 80
E1-Nasotype
E2-Nasotype
E3-Nasotype
-1.0 -0.5 0 0.5 1.0
Spearman r
D) ALLIANCE adult asthma arm: age group ≥65
correlation (r)
N = 50
E1-Nasotype
E2-Nasotype
E3-Nasotype
-1.0 -0.5 0 0.5 1.0
Spearman r
E) ALLIANCE adult asthma arm
gene expression
18-39
40-64
≥65
age group
E1-Nasotype
E2-Nasotype
E3-Nasotype
Color range
-1.5 -0.8 0 0.8 1.5
normalized intensity values

Figure S3

A) URECA cohort

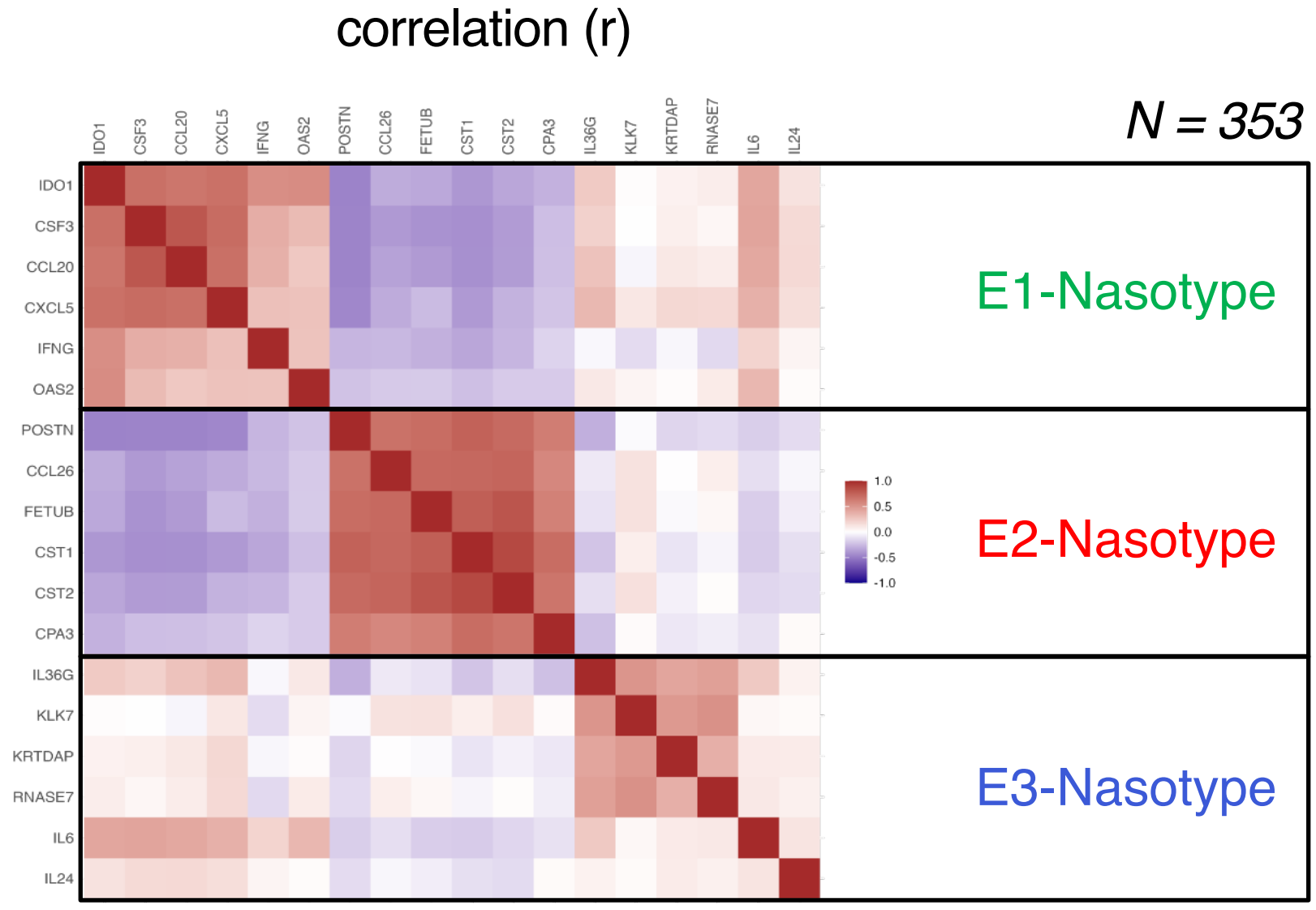

B) GRASS cohort

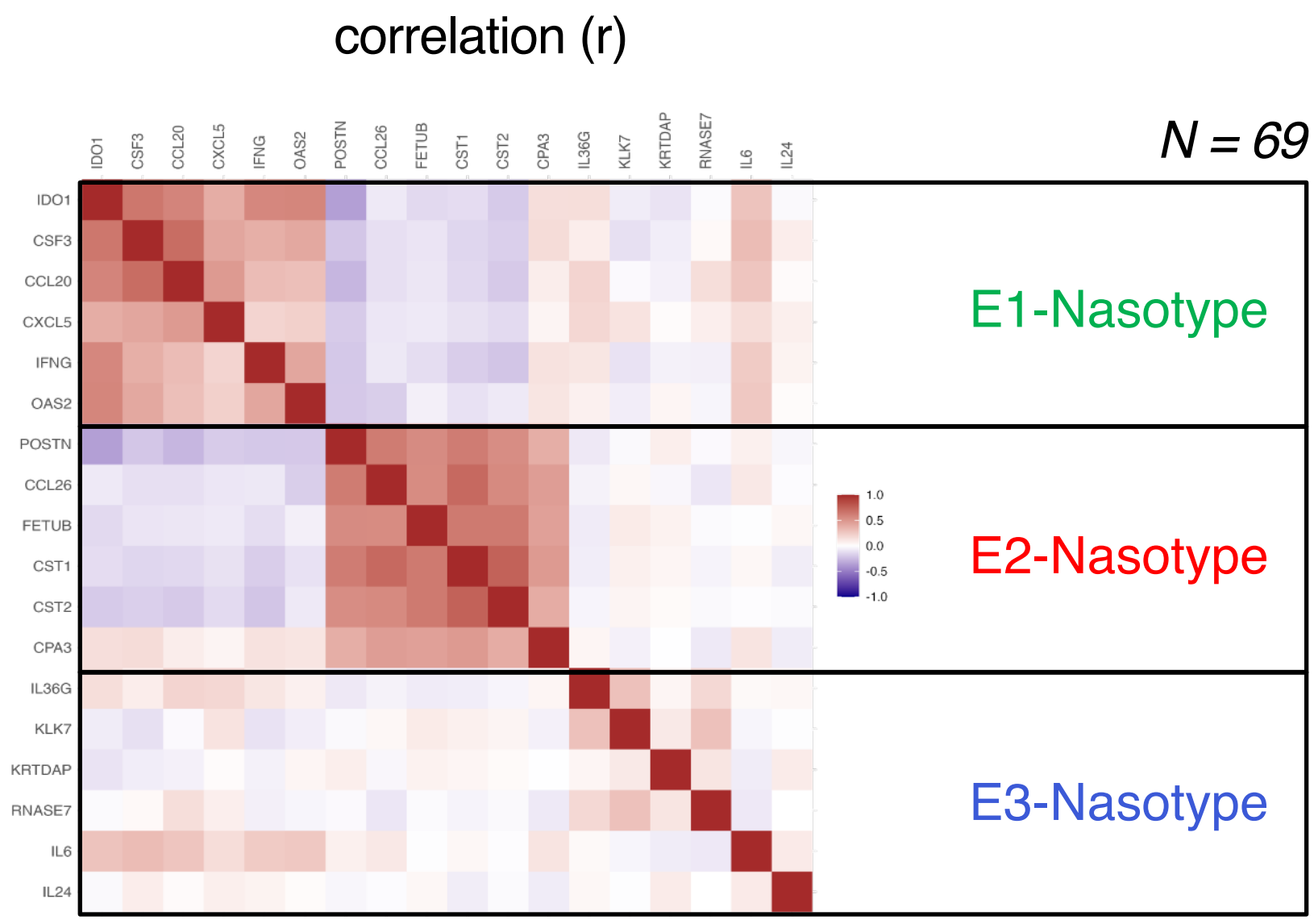

C) CatEEC cohort

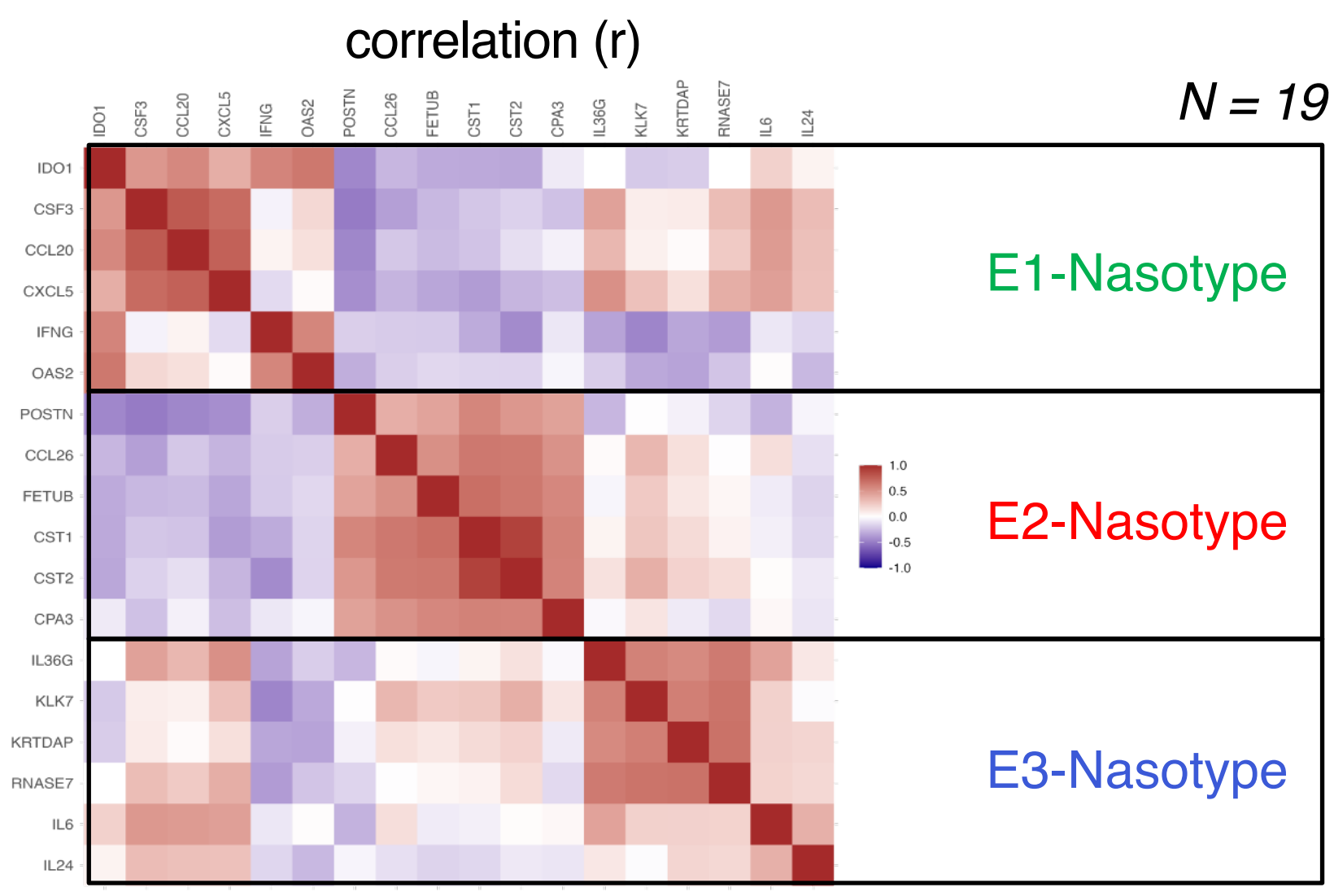

Figure S4

A

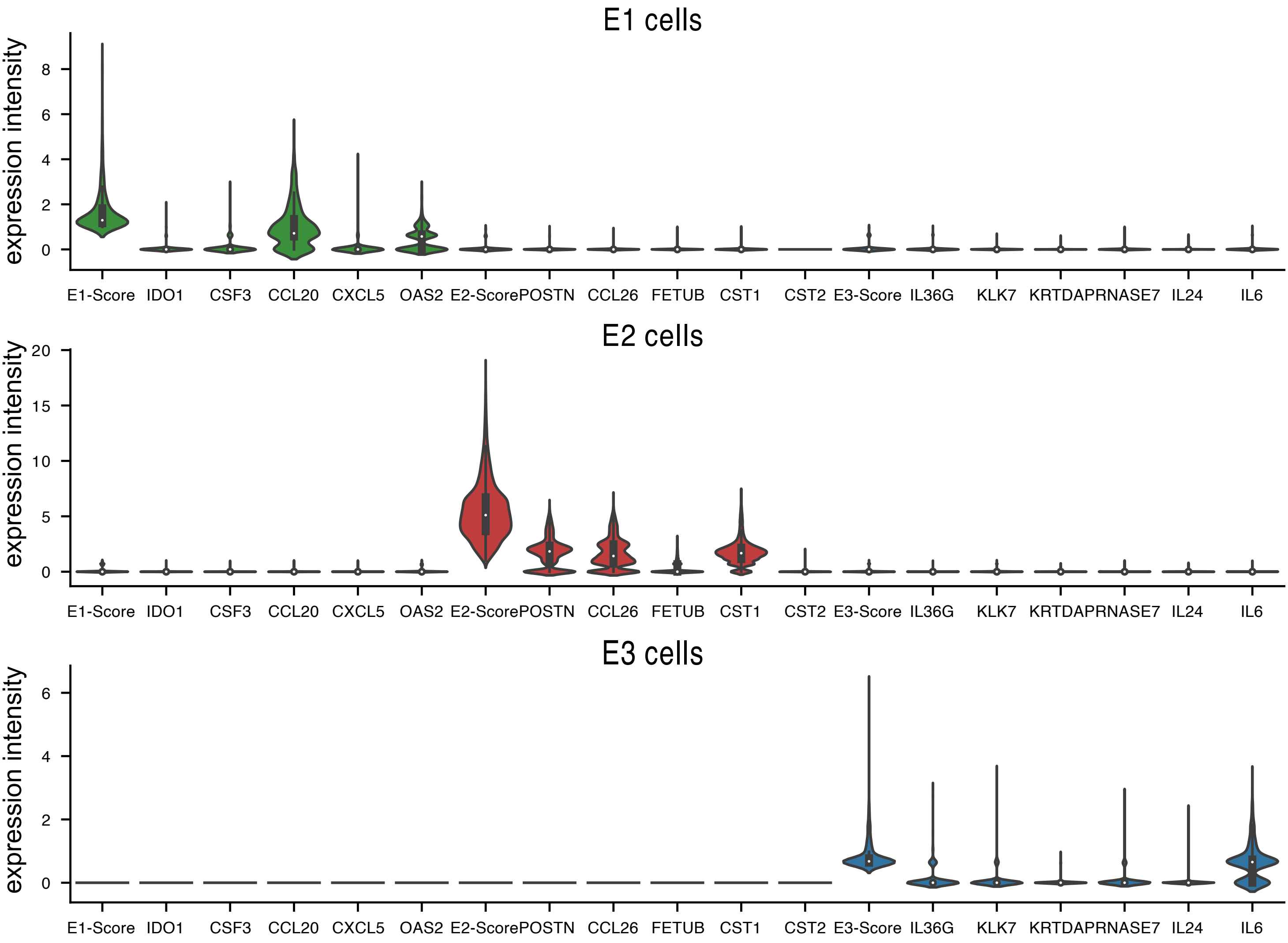
E1 cells
expression intensity
E1-Score
IDO1
CSF3
CCL20
CXCL5
OAS2
E2-Score
POSTN
CCL26
FETUB
CST1
CST2
E3-Score
IL36G
KLK7
KRTDAP
RNASE7
IL24
IL6
E2 cells
E3 cells

B

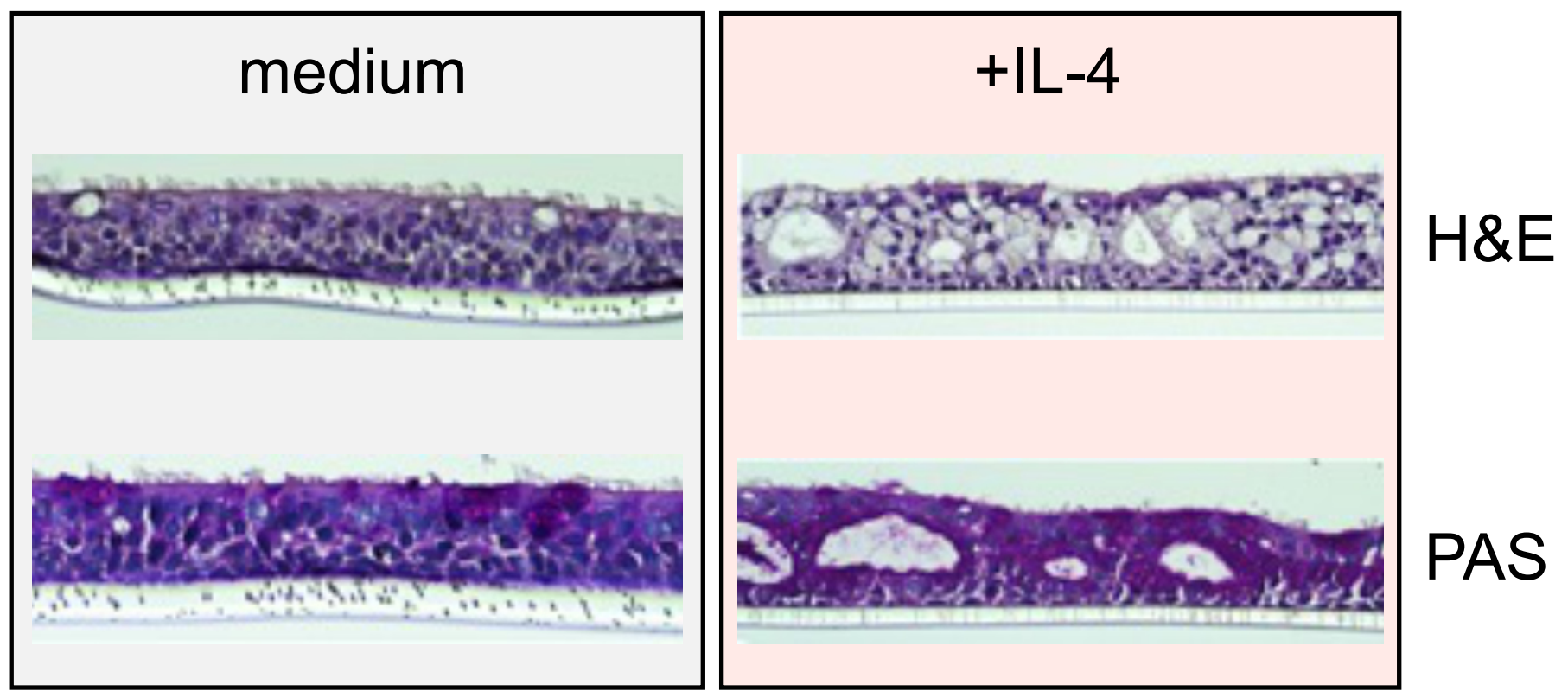
medium
+IL-4
H&E
PAS

Figure S5

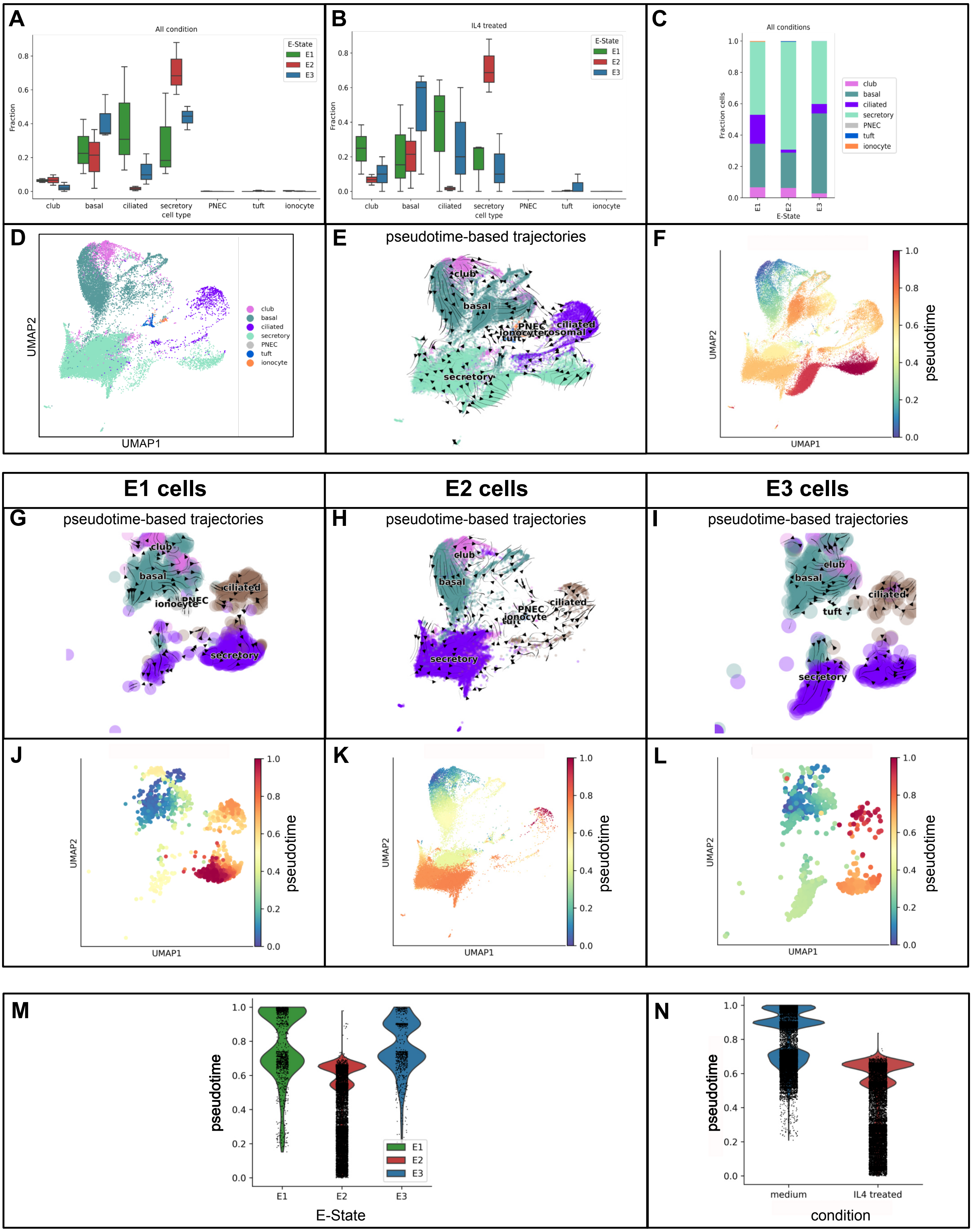
A
All condition
Fraction
E-State
E1
E2
E3
club
basal
ciliated
secretory
PNEC
tuft
ionocyte
cell type
B
IL4 treated
C
All conditions
Fraction cells
E-State
D
UMAP1
UMAP2
E
pseudotime-based trajectories
F
pseudotime
E1 cells
E2 cells
E3 cells
G
H
I
J
K
L
M
E-State
N
medium
IL4 treated
condition

Figure S6

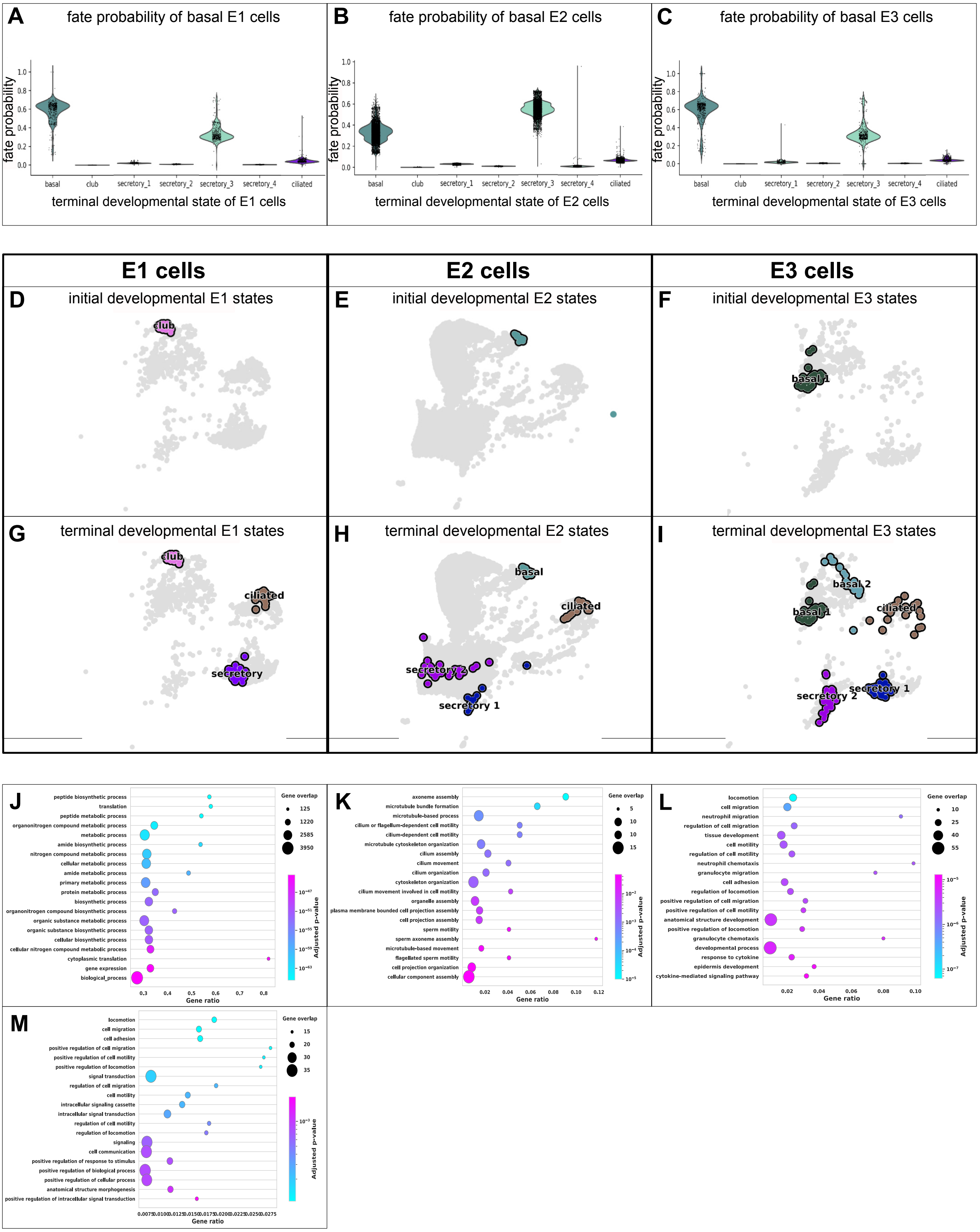
A
fate probability of basal E1 cells
fate probability
terminal developmental state of E1 cells
B
fate probability of basal E2 cells
fate probability
terminal developmental state of E2 cells
C
fate probability of basal E3 cells
fate probability
terminal developmental state of E3 cells
basal
club
secretory_1
secretory_2
secretory_3
secretory_4
ciliated
E1 cells
E2 cells
E3 cells
D
initial developmental E1 states
club
E
initial developmental E2 states
F
initial developmental E3 states
basal 1
G
terminal developmental E1 states
club
ciliated
secretory
H
terminal developmental E2 states
basal
ciliated
secretory 2
secretory 1
I
terminal developmental E3 states
basal 2
basal 1
ciliated
secretory 2
secretory 1
J
K
L
M
Gene overlap
Adjusted p-value
Gene ratio

Figure S7

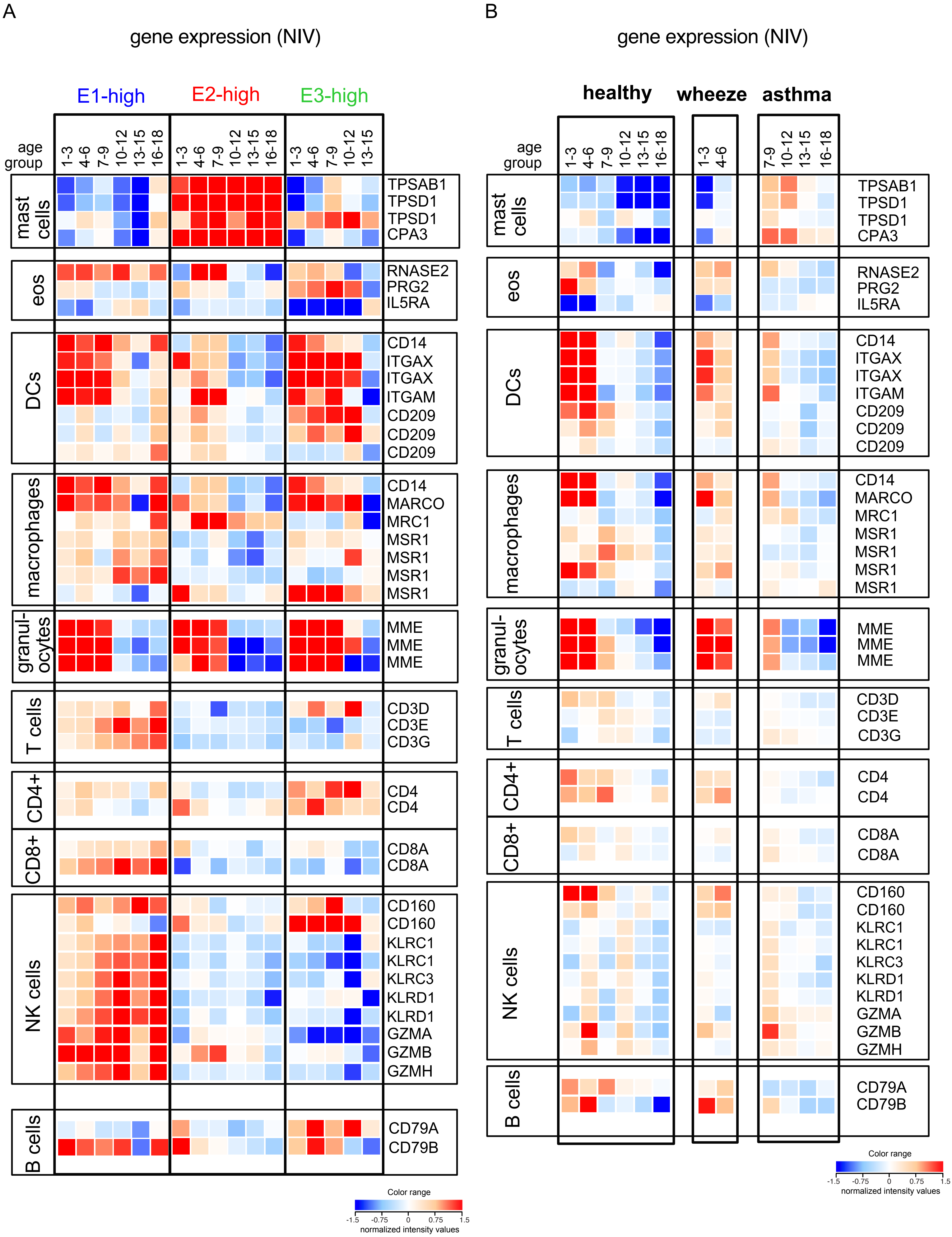
A
gene expression (NIV)
E1-high
E2-high
E3-high
age group
1-3
4-6
7-9
10-12
13-15
16-18
mast cells
eos
DCs
macrophages
granul-ocytes
T cells
CD4+
CD8+
NK cells
B cells
TPSAB1
TPSD1
TPSD1
CPA3
RNASE2
PRG2
IL5RA
CD14
ITGAX
ITGAX
ITGAM
CD209
CD209
CD209
CD14
MARCO
MRC1
MSR1
MSR1
MSR1
MSR1
MME
MME
MME
CD3D
CD3E
CD3G
CD4
CD4
CD8A
CD8A
CD160
CD160
KLRC1
KLRC1
KLRC3
KLRD1
KLRD1
GZMA
GZMB
GZMH
CD79A
CD79B
Color range
-1.5
-0.75
0
0.75
1.5
normalized intensity values
B
gene expression (NIV)
healthy
wheeze
asthma
age group
1-3
4-6
7-9
10-12
13-15
16-18
mast cells
eos
DCs
macrophages
granul-ocytes
T cells
CD4+
CD8+
NK cells
B cells
TPSAB1
TPSD1
TPSD1
CPA3
RNASE2
PRG2
IL5RA
CD14
ITGAX
ITGAX
ITGAM
CD209
CD209
CD209
CD14
MARCO
MRC1
MSR1
MSR1
MSR1
MSR1
MME
MME
MME
CD3D
CD3E
CD3G
CD4
CD4
CD8A
CD8A
CD160
CD160
KLRC1
KLRC1
KLRC3
KLRD1
KLRD1
GZMA
GZMB
GZMH
CD79A
CD79B
Color range
-1.5
-0.75
0
0.75
1.5
normalized intensity values

Figure S8 - U-BIOPRED: TAC1, TAC2, TAC3 phenotypes

Correlation (Spearman r)

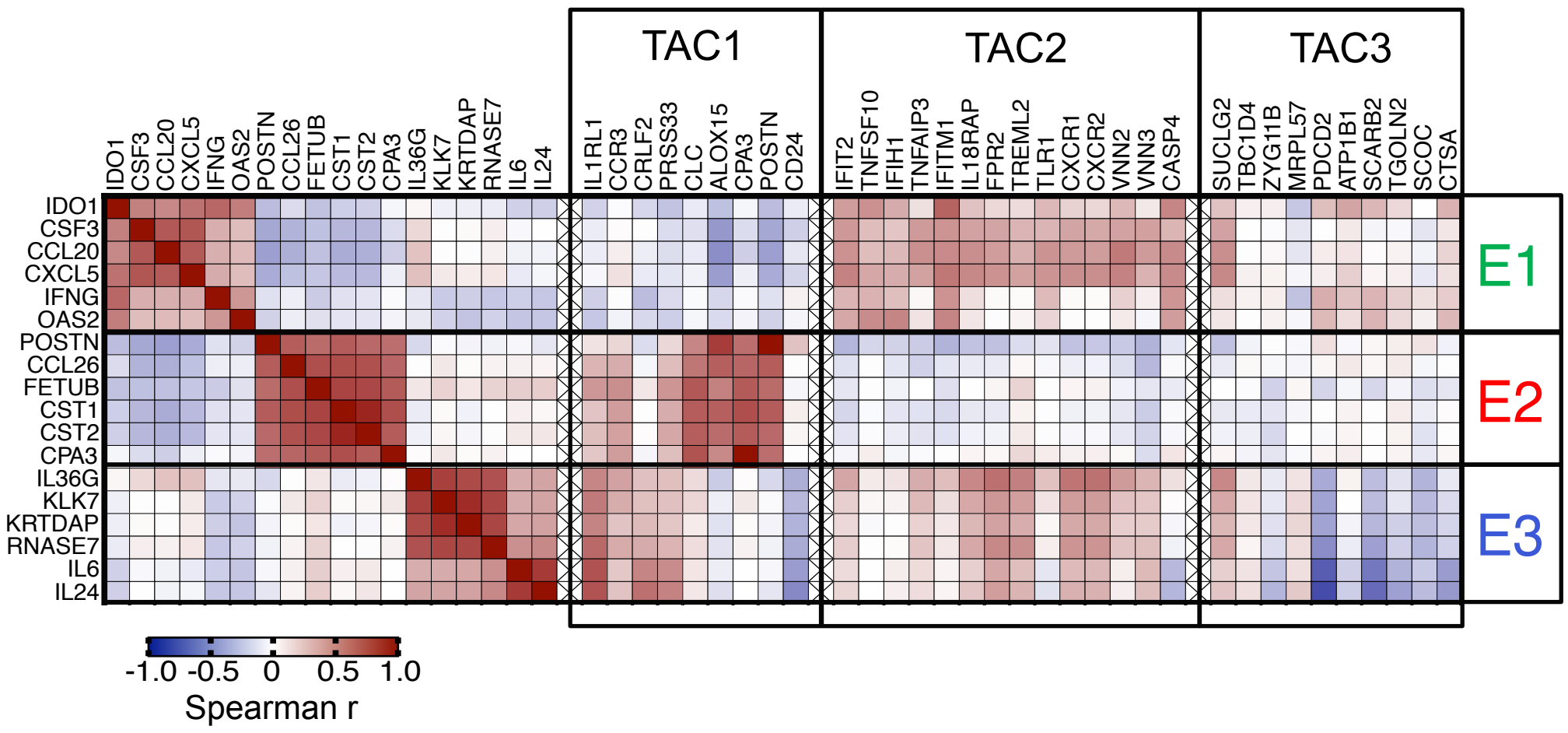

Supplement: Supplementary file 1 [file ALL-81-3533-s002.pdf]
