# Supplementary material for: Nasal Epithelial Immune Signatures Are Associated With Age‐Dependent Asthma Trajectories in Early Life
Source: Allergy. 2026 Sep 6;81(10):3533–53. doi: 10.1111/all.70477 (PMC13646873; doi:10.1111/all.70477)
Supplement: Supplementary file 2 [file ALL-81-3533-s001.docx]

**Nasal epithelial immune signatures are associated with age-dependent asthma trajectories in early life**

Constanze A. Jakwerth^1,2^, Sabina Illi^2,3,5^, Helen Charles^1,2^, Madlen Oelsner^1,2^, Amr A. Gabr^1,2^, Ulrich M. Zissler^1,2^, Anna Erb^1,2^, Martin Feuerherd^18,19^, Felix Drost^20,21^, Maren Büttner^20^, Benjamin Schubert^2,20,22^, Nicole Maison^2,3,4,5^, Jimmy Omony^2,3,5^, Markus Weckmann^2,6,7,10^, Ruth Grychtol^2,11^, Anna-Maria Dittrich^2,11^, Svenja Foth^2,15^, Chrysanthi Skevaki^2,14^, Tobias Trojan^2,16^, Silke van Koningsbruggen-Rietschel^2,16^, Gesine Hansen^2,11,17^, Thomas Bahmer^2,10,12^, Klaus F. Rabe^2,10,13^, Folke Brinkmann^2,6,7,10^, Matthias Volkmar Kopp^7,9,10^, Ulrike Protzer^18,19^, Adam M. Chaker^1,8^, Bianca Schaub^2,4,5,23^, Erika von Mutius^2,3,4,5^, Carsten B. Schmidt-Weber^1,2^ and the ALLIANCE Study Group as part of the German Center for Lung Research (DZL)^24^

1) Center of Allergy and Environment (ZAUM), School of Medicine and Health, Technical University of Munich, and Helmholtz Munich, Munich, Germany

2) Member of the German Center for Lung Research (DZL), Germany

3) Institute for Asthma and Allergy Prevention (IAP), Helmholtz Munich, German Research Center for Environmental Health (GmbH), Munich, Germany

4) Dept. of Pulmonary and Allergy, Dr von Hauner Children's Hospital, University Children´s Hospital, Ludwig Maximilians University, Munich, Germany

5) Comprehensive Pneumology Center - Munich (CPC-M), Member of German Center for Lung Research (DZL), Munich, Germany

6) Division of Epigenetics in Chronic Lung Disease, Priority Area Chronic Lung Diseases, Research Center Borstel- Leibniz Lung Center, Borstel, Germany

7) Division of Pediatric Pneumology and Allergology, University Children’s Hospital Lübeck, University Hospital Schleswig-Holstein, Campus Lübeck, Lübeck, Germany

8) Department of Otorhinolaryngology, TUM School of Medicine and Health, Klinikum rechts der Isar, Technical University of Munich, Munich, Germany

9) University Children’s Hospital, Inselspital, Bern University Hospital, University of Bern, Bern, Switzerland

10) Airway Research Center North (ARCN), Germany

11) Department of Paediatric Pneumology, Allergology and Neonatology, Hannover Medical School, Hannover, Germany, and Biomedical Research in Endstage and Obstructive Lung Disease Hannover (BREATH), Germany

12) University Hospital Schleswig-Holstein - Campus Kiel, Internal Medicine Department I, Kiel, Germany

13) LungenClinic Grosshansdorf GmbH and Medical Clinics, Christian Albrechts University, Kiel, Germany

14) Institute of Laboratory Medicine and Pathobiochemistry, Molecular Diagnostics, University of Marburg, Germany, and University of Gießen, Marburg Lung Center (UGMLC)

15) Universities of Giessen and Marburg Lung Center (UGMLC), Philipps University Marburg, Marburg, Germany; University Children's Hospital Marburg, University of Marburg, Marburg, Germany

16) Pediatric Pulmonology and Allergology, Department of Pediatrics, University of Cologne, Faculty of Medicine and University Hospital Cologne, Cologne, Germany

17) Cluster of Excellence RESIST (EXC 2155), Hannover Medical School, Carl-Neuberg-Straße 1, 30625 Hannover, Germany

18) Institute of Virology, School of Medicine and Health, Technical University of Munich / Helmholtz Munich, Munich, Germany.

19) German Center for Infection Research (DZIF), Munich partner site, Munich, Germany

20) Institute of Computational Biology, Helmholtz Munich—German Research Center for Environmental Health, Neuherberg, Germany

21) School of Life Sciences Weihenstephan, Technical University of Munich, Munich, Germany

22) Department of Mathematics, Technical University of Munich, Garching bei München, Germany

23) German Center for Child and Adolescent Health (DZKJ), Dr von Hauner Children's Hospital, LMU Munich, Germany

24) Members of the ALLIANCE Study Group are listed in the Acknowledgements.

* Corresponding author:	Prof. Dr. Carsten B. Schmidt-Weber

Center of Allergy and Environment (ZAUM),

Technische Universität and Helmholtz Center Munich,

Biedersteiner Str. 29

80802 München, Germany

[csweber@tum.de](mailto:csweber@tum.de)

phone +49 89 41403450

**SUPPLEMENTARY METHODS**

***Ethics***

The ALLIANCE cohort (German Center for Lung Research, DZL) prospectively follows children and adults with wheeze/asthma (paediatric arm: ClinicalTrials.gov [NCT02496468](http://clinicaltrials.gov/show/NCT02496468); adult arm: [NCT02419274](http://clinicaltrials.gov/show/NCT02419274)). The responsible Ethics Committees approved the study protocol at all participating centers, including the coordinating ethics board at Ludwig-Maximilians-University Munich (approval no. 516-12) and the local committees at Hannover Medical School, University of Lübeck, and University Hospital of Cologne. All procedures were performed in accordance with the Declaration of Helsinki and Good Clinical Practice (ICH-GCP) guidelines. Written informed consent was obtained from the parents or legal guardians of all participating children, and where appropriate, assent was obtained from the children themselves. Adult participants provided written informed consent prior to enrolment.

***Nasal gene expression analysis***

Nasal epithelial samples were collected from children using interdental brushes (IDB-G50, Top Caredent). The medial and superior surface of the inferior nasal meatus was brushed with gentle rotary and linear movements and immediately immersed in sterile phosphate-buffered saline. The brush head was transferred into RNAprotect Cell Reagent (Qiagen) in a pre-cooled 2-mL cryotube, trimmed to fit, and stored at -80 °C until processing. For each participant, only the earliest available nasal brushing was included in the analysis. Peripheral blood was collected in K⁺-EDTA-coated S-Monovettes (Sarstedt) and frozen for subsequent DNA extraction and genotyping.

Total RNA was extracted using the AllPrep DNA/RNA Micro Kit (Qiagen) according to the manufacturer’s instructions. RNA concentration and purity were determined by UV-spectrophotometry (NanoDrop Technologies), and integrity was assessed with the RNA 6000 Nano Chip Kit on an Agilent 2100 Bioanalyzer (Agilent Technologies). Amplification and Cy3-labeling were performed with the one-color Quick Amp Labeling Kit (Agilent Technologies), followed by hybridization to SurePrint G3 Human Gene Expression 8×60K microarrays (Agilent Technologies) using the Gene Expression Hybridization Kit.

Raw data were processed in GeneSpring GX v14.9.1 (Agilent Technologies). Quality control included verification of spike-in controls and exclusion of compromised probes. Expression data were normalized using GeneSpring’s 80th-percentile normalization. Differential expression between groups was determined by moderated *t*-test with Benjamini-Hochberg correction (fold change ≥ 1.5, P < 0.05, FDR < 5%). Normalized expression values of genes encoding secreted products defined by Gene Ontology (GO) terms “cell-cell signalling” (GO:0007267), “cytokine activity” (GO:0005125), “chemokine activity” (GO:0008009), or “extracellular space” (GO:0005615), as well as selected genes of interest detectable in nasal secretions (*IDO1, FETUB, OAS2, PRB2*), were analysed by Spearman’s rank correlation (complete candidate transcript list provided in Tab. S31). To identify epithelial immune programs, candidate transcripts from this candidate list of epithelial secreted/immune-related transcripts were ranked by Spearman correlation with predefined cytokine seed variables: IFNG for type-1 responses, mean(IL4, IL13) for type-2 responses, and mean(IL17A, IL36G) for type-3 responses. Final module genes were selected from the top-ranked candidates based on both their association with the respective seed variable and their positive correlations within the respective module. This approach defined three epithelial immune indices: E1 (type-1; *IDO1, CSF3, CCL20, CXCL5, IFNG, OAS2*), E2 (type-2; *POSTN, CCL26, FETUB, CST1, CST2, CPA3*), and E3 (type-3; *IL36G, KLK7, KRTDAP, RNASE7, IL24, IL6*). Each index was calculated as the mean normalized expression of its respective marker genes.

***Cytokine stimulation of NHBEs***

Primary normal human bronchial epithelial cells (NHBE, Lonza) derived from genetically independent donors were cultured as monolayers in serum-free bronchial epithelial cell growth medium (BEGM BulletKit, Lonza) at low passage numbers. Prior to stimulation, NHBEs were starved overnight in BEBM medium (BEGM without supplements, Lonza). Cells were then stimulated with IFN-γ (100 IU/mL, Biotechne), IL-4 (50 ng/mL, Biotechne), or IL-17A (100 ng/mL, Biotechne) alone, or in combination with IL-36γ (100 ng/mL, Biotechne), for 6 or 24 hours as indicated. After stimulation, cells were lysed in RLT buffer (Qiagen) for RNA isolation and quantitative PCR (qPCR; Roche) analysis. Cell culture supernatants were collected and stored for subsequent measurement of secreted mediators.

***Cytokine-induced epithelial programs in air-liquid interface cultures***

NHBEs from three genetically independent donors at low passage were expanded in PneumaCult-Ex Plus medium (Stemcell) and seeded on collagen-coated polyester transwell inserts (12 mm diameter, 0.4 μm pore size, type 3460; Merck). Inserts were precoated with 1% collagen (Sigma-Aldrich) for 30 minutes at 37 °C. At 95% confluence (day 0), NHBEs were transferred to the pre-coated inserts at a density of 3 × 10⁵ cells/cm² in PneumaCult-Ex Plus medium. Initially, 1 mL of medium was added to the basal and 0.5 mL to the apical compartment. On day 3, cells were lifted to the air-liquid interface by removing the apical medium, and the basal medium was replaced by PneumaCult-ALI maintenance medium (Stemcell). Transepithelial electrical resistance (TEER) was measured using an EVOM2 instrument (World Precision Instruments). When epithelial layers had formed dense, tissue-like structures with TEER values of ≥ 600 Ω·cm², hydrocortisone was withdrawn and ALI cultures were stimulated from the basal side with IFNγ (100 IU/mL), IL-4 (50 ng/mL), or IL-17A (100 ng/mL) plus IL-36γ (100 ng/mL). Cytokines were refreshed every other day throughout the differentiation period. The IL-4 concentration is commonly used in epithelial polarization assays^1,2^ and was selected to induce robust type-2 programming during long-term ALI differentiation under conditions of sustained IL-4Rα stimulation (KD ≈ 10⁻¹⁰ M ^3^). IL-4 was deliberately selected as the sole type-2 stimulus since it binds IL-4Rα with high affinity and can engage both type-I and type-II IL-4 receptor complexes, whereas IL-13 signals only through the type-II receptor. In airway epithelial cells, both cytokines converge on IL-4Rα/IL-13Rα1-STAT6 signaling. Cultures were harvested for single-cell RNA-sequencing when fully differentiated, defined by TEER values >700 Ω·cm², typically after ≥21 days at the air-liquid interface using TrypLE Express Enzyme (Thermo Fisher Scientific) according to the manufacturer’s instructions. For microscopy, ALI cultures were fixed in 4% paraformaldehyde (30 min, RT) and stored in 3.7% formaldehyde until paraffin embedding. Three-micrometer FFPE sections were prepared using an RM2255 microtome (Leica) and subjected to standard hematoxylin-eosin (H&E) and periodic acid-Schiff (PAS) staining to assess epithelial morphology.

***Single-cell mRNA-sequencing analysis of 3D air-liquid interface (ALI) cultures***

Single-cell 5′ gene expression libraries were prepared using the Chromium Next GEM Single Cell 5′ Library Kit v1.1 and Gel Bead Kit v1.1 (10x Genomics) on the Chromium Controller instrument (10x Genomics) following the manufacturer’s protocol. Library quality and fragment size were verified using the High Sensitivity DNA Analysis Kit on a Bioanalyzer (Agilent) and quantified with the KAPA Library Quantification Kit (Roche). Libraries passing quality criteria were sequenced on a NovaSeq 6000 platform (Illumina) at the Helmholtz Center Munich sequencing facility, yielding on average 11,378 cells per sample and 62,930 reads per cell.

Raw sequencing data were processed with CellRanger v3.1.0 (10x Genomics) and aligned to the human reference genome hg38. Barcodes, UMIs, and gene counts were extracted, and cells expressing fewer than 200 genes, with fewer than 500 UMIs, or with >20 % mitochondrial reads were excluded. Genes expressed in fewer than 10 cells were removed. Doublets were detected using scrublet v0.2.3 ^4^; cells with a doublet score > 0.2 were discarded. After stringent filtering, six samples (three replicates per condition) comprising 56,807 cells and 16,478 genes were retained.

Normalization and batch correction were performed in Scanpy v1.5.1 ^5^ using a two-step procedure: (i) library-size normalization to the median total UMI count per cell, followed by recalculation of Scran size factors; (ii) log1p transformation. Dimensionality reduction was achieved by principal component analysis (PCA; 50 components). The number of principal components was chosen to capture epithelial differentiation gradients across donors and conditions; clustering robustness and annotation were verified using canonical epithelial markers (Fig. S5). The 3,000 most variable genes were selected based on mean expression and dispersion using the “cell_ranger” method. Neighborhood graphs (30 nearest neighbors per cell) were computed from the first 50 principal components. Cell clustering employed the Louvain algorithm at a resolution of 0.5. Batch effects were corrected by scaling the normalized expression matrix to zero mean and unit variance per gene, followed by Scanorama v1.7 ^5^ integration. Final embeddings were visualized using UMAP ^6^.

Cell clustering and annotation were refined with the Leiden algorithm ^7^ ^8^ applied to each sample separately. Cell identities were assigned based on canonical epithelial marker genes: basal (*KRT6A, VWF, DLK2*), ciliated (*FOXJ1, TUBA1A, STK11*), secretory (*SCGB1A1, KRT4, KRT13, YAP1, CEACAM5, CEACAM6, MUC5AC, MUC5B, SPDEF, FOXA3*), club (*MUC2, CCL26*), ionocytes (*CFTR, FOXI1*), tuft (*DCLK1, AVIL*), and pulmonary neuroendocrine cells (*CALCA, SST, CHGA, GRP*). Immune cells (*CD3G, PTPRC*) were identified and excluded. Ambiguous clusters co-expressing multiple lineage markers were re-clustered at resolutions 0.5 or 1.0 until homogeneous expression patterns were achieved, and final annotations were merged across replicates.

Epithelial immune-state subsets (E1, E2, E3) were defined based on expression of previously described marker genes: E1 (*IDO1, CSF3, CCL20, CXCL5, OAS2*), E2 (*POSTN, CCL26, FETUB, CST1, CST2*), E3 (*IL36G, KLK7, KRTDAP, RNASE7, IL24, IL6*). E-state scores were calculated for each cell as the sum of log-transformed marker gene expression values. Cells were assigned to the E1, E2, or E3 state if the corresponding score exceeded 1.0, 1.0, and 0.5, respectively.

Trajectory inference was performed using Palantir v1.3.3 ^9^ to reconstruct pseudo-time ordering across all cells and within each E-state subset. A basal cell with the minimal diffusion component served as the starting point. Palantir was run with 500 sampled waypoints under default parameters. CellRank2 v2.0.6 ^10^ was then applied to infer macro-states, identify initial and terminal states, and compute fate probabilities for each cell.

Differential gene expression between epithelial immune states (E2 vs. E1 and E2 vs. E3) was analyzed for each epithelial subtype using the Limma package v3.38.3 in R v3.5.1, applying Benjamini-Hochberg correction for multiple testing. Gene expression patterns were visualized using Scanpy v1.5.1 (Python 3.8). Gene set enrichment analysis (GSEA) was performed with the g:Profiler tool using the official gprofiler-official Python package v1.0.0, with all expressed genes serving as the background set. Gene sets with an adjusted p-value ≥ 0.05 were excluded. To distinguish activation from repression programs, input gene lists were divided by the direction of log-fold change, and upregulated and downregulated genes were analyzed separately within each contrast.

For the additional cytokine-stimulation experiment (Fig. 3D-G), ALI-differentiated NHBE cultures stimulated with IFNγ, IL-4, or IL-17A+IL-36γ were analysed by scRNA-seq. Raw sequencing data were processed using Cell Ranger v9.0.1 (10x Genomics) and aligned to the GRCh38-2024-A reference transcriptome. The dataset comprised five independent donor samples for medium, IFNγ and IL-4 conditions, two of which were also stimulated with IL-17A+IL-36γ. Cells were retained if they contained at least 150 detected genes, at least 400 UMIs, and ≤25% mitochondrial reads. Analysis was performed in R 4.6.0 (2026-04-24) using Seurat 5.5.0, SeuratObject 5.4.0, Matrix 1.7.5, ggplot2 4.0.3, dplyr 1.2.1, tidyr 1.3.2, readr 2.2.0, patchwork 1.3.2, and pheatmap 1.0.13. Samples were log-normalized, variable features were identified independently for each sample, and datasets were integrated using reciprocal PCA-based anchor integration. The integrated object was scaled and used for principal component analysis (PCA), graph-based clustering, and UMAP visualization. Cluster marker genes were identified using FindAllMarkers. E1, E2, and E3 epithelial response program scores were calculated using predefined marker gene sets as described above.

***Validation of correlation analyses in published nasal transcriptome datasets from independent cohorts***

To assess the preservation of nasotype gene-gene correlation structure beyond the ALLIANCE cohort and mitigate concerns of circularity, publicly available nasal transcriptome datasets were analysed, including the Urban Environment and Childhood Asthma (URECA) cohort (^11^; GEO: GSE145505), the GRASS allergic rhinitis cohort (^12^; GEO: GSE206151), and the Cat dander Environmental Exposure Chamber (CatEEC) cohort (^13^; GEO: GSE129748). Analyses were conducted in R (v4.4.0). Gene count and sample metadata were filtered and normalized using the DESeq function ^14^. Counts were converted to transcripts per million (TPM) and log-scaled (LSTPM) to enable comparability of expression levels within each dataset. Pairwise gene correlations were computed using the Hmisc package (v5.1-3), and correlation matrices were visualized with ComplexHeatmap ^15^. For external replication cohorts, within-module correlation strengths were summarized as the median Spearman correlation coefficient across all gene pairs within each module.

***Statistical analyses***

Multivariate linear regression models were used in the pediatric arm of the ALLIANCE cohort to assess associations between E1, E2, and E3 gene expression indices and population characteristics, both in the total population and stratified by disease status. All models were adjusted for age, and models in the total population were additionally adjusted for disease status, as these variables were significantly associated with E-state expression and thus considered potential confounders. Interaction terms for disease status were tested within full hierarchical models.

To assess the association between early nasotype activity and asthma persistence, we fitted multivariable logistic regression models in preschool wheezers with asthma status at age 6 years (yes/no) as the dependent variable. Independent variables were the continuous E1, E2 and E3 nasotype indices. Separate univariate models and combined multivariable models were calculated. Multivariable models were adjusted a priori for age at enrolment and atopic sensitization (RAST class ≥ II), and symptoms of acute ENT infection based on established clinical relevance. A p-value < 0.05 was considered statistically significant. All regression analyses were performed in SAS (v9.4, SAS Institute). Dataset version 5 (V5) was used for all analyses. Formal sensitivity analyses were not conducted because the multivariate models were prespecified based on biological hypotheses and validated across independent cohorts. This a priori modeling approach was chosen to minimize overfitting and increase generalizability.

Nasotype indices were predefined based on gene correlation structure and were not derived from outcome-based training. Accordingly, analyses reflect signature-based stratification and association testing rather than the development of a predictive classifier.

Differential nasal gene expression between groups was determined by moderated *t*-test with Benjamini-Hochberg correction for multiple testing (fold change ≥ 1.5, P < 0.05, FDR < 5%).

Group comparisons of nasotype activity indices (E-indices) were performed using two-sided Mann-Whitney *U* tests (GraphPad Prism v10.4.1). Increasing trends in predefined marker-gene expression across Q1-Q5 were assessed in R version 4.6.0 (2026-04-24) using one-sided Jonckheere-Terpstra tests with asymptotic P values. P values were adjusted separately within each nasotype module using the Benjamini-Hochberg method, and Spearman’s rho was calculated as a descriptive measure of monotonic association. Violin plots display all data points with medians indicated; significance levels are denoted as *p<0.05, **p<0.01, and ***p<0.001.

Correlations between model gene expression levels were evaluated by Spearman’s rank correlation and simple linear regression (GraphPad Prism v10.4.1); regression lines depict the mean with 95% confidence intervals.

**SUPPLEMENTARY FIGURE LEGENDS**

**Supplementary Figure 1: Study populations included in the discovery and replication analyses.** Flowchart of sample selection in the paediatric and adult ALLIANCE cohorts. The source cohort comprised 561 paediatric and 224 adult samples; 18 siblings were excluded from the paediatric arm. Nasal transcriptome analyses included 265 genetically independent children and 174 adults. Independent replication cohorts comprised URECA (N=353), GRASS (59 participants; 256 samples), and CatEEC (19 participants; 256 samples).

**Supplementary Figure 2: Validation of E1-E3 expression programs in adults with asthma from the ALLIANCE cohort.** Spearman correlation matrices of the six highest-ranked E1-, E2-, and E3-associated genes in all genetically independent adults with asthma (A; N=149) and in participants aged 18-39 years (B; N=19), 40-64 years (C; N=80), and ≥65 years (D; N=50). Blue and red indicate negative and positive correlations, respectively. (E) Normalized gene-expression intensity in adults with asthma stratified by age group. The colour scale ranges from −1.5 to 1.5 normalized intensity values. Asterisks indicate differential expression between participants aged ≥65 and 18-39 years (moderated t-test, p<0.05 and fold change ≥1.5).

**Supplementary Figure 3: Replication of E1-E3 gene-expression programs in independent nasal transcriptome cohorts.** Spearman correlation matrices of E1-, E2-, and E3-associated genes in URECA (A; N=353; one sample per participant; GSE145505), GRASS (B; 256 samples from 59 participants; GSE206151), and CatEEC (C; 256 samples from 19 participants; GSE129748). The colour scale ranges from Spearman’s ρ=−1 to ρ=1.

**Supplementary Figure 4. E1-E3 expression and epithelial morphology in differentiated airway epithelial cultures.** (A) Expression of E1-, E2-, and E3-associated genes in the corresponding single-cell E states, shown as violin plots of transcript copy numbers. (B) Representative haematoxylin and eosin and periodic acid-Schiff staining of differentiated NHBE air-liquid interface cultures maintained with or without IL-4. Images were acquired at 40× magnification and represent three independent cultures.

**Supplementary Figure 5: Developmental trajectories of E1, E2, and E3 epithelial states**. Distribution of E1, E2, and E3 cells across epithelial cell types under all treatment conditions (A) and after IL-4 treatment (B). (C) Cell-type composition within each E state. (D) UMAP representation of NHBE cells differentiated for 12 days at air-liquid interface under medium or IL-4 conditions (50 ng/mL, added basolaterally every 48 h). Basal, ciliated, club, secretory, pulmonary neuroendocrine, tuft, and ionocyte populations are indicated. Palantir pseudotime trajectories are shown for all cells (E), E1 cells (G), E2 cells (H), and E3 cells (I), with corresponding coarse-grained transition matrices in F and J-L. Colours indicate progression from early to late pseudotime. Pseudotime distributions are shown by E state (M) and treatment condition (N).

**Supplementary Figure 6: Cell-fate probabilities and pathway enrichment across E1-E3 epithelial trajectories.** Violin plots show cell-fate probabilities of basal E1 (A), E2 (B), and E3 (C) cells for differentiation towards basal, club, four secretory, and ciliated cell states. Initial states (D-F) and terminal states (G-I) are shown for the corresponding E1-E3 trajectories. Gene-set enrichment analyses compare secretory E1 versus E2 cells (J, K) and basal E1 or E3 versus E2 cells (L, M).

**Supplementary Figure 7. Age-resolved leukocyte-marker expression across epithelial nasotypes and disease groups.** (A) Leukocyte cell-type marker expression in 265 genetically independent children with high E1, E2, or E3 index expression, defined as quintile 5, and stratified into age groups of 1-3, 4-6, 7-9, 10-12, 13-15, and 16-18 years. (B) Corresponding age-resolved expression in healthy children and children with wheeze or asthma. Heatmaps show normalized intensity values ranging from −1.5 to 1.5.

**Supplementary Figure 8: Correlations between epithelial nasotypes and U-BIOPRED transcript-associated clusters.** Spearman correlation matrix of the six highest-ranked E1-, E2-, and E3-associated genes and U-BIOPRED transcript-associated clusters. (A) Correlation coefficients, with blue and red indicating negative and positive correlations, respectively. (B) Corresponding p values, with shading ranging from light grey for p<0.05 to dark grey for p<0.0001.

**SUPPLEMENTARY TABLES**

All supplementary tables can be found in the attached SuppTables.xlsx file.

Supplementary Table S1-1: Correlation matrix of nasotypes from all paediatric samples (N=265) - Spearman r values; Supplementary Table S1-2**.** Correlation matrix of nasotypes from all paediatric samples (N=265) - Spearman P values

Supplementary Table S2: Seed correlation ranking and within-module correlations of final nasotype marker genes

Supplementary Table S3: Ordered trends of predefined E1-, E2-, and E3-associated genes across their respective cytokine seed-variable quintiles

Supplementary Table S4-1. Correlation matrix of nasotypes from all adult samples of the ALLIANCE cohort (N=149) - Spearman r values; Supplementary Table S4-2. Correlation matrix of nasotypes from adult samples of age group 18-39 (N=19) - Spearman r values; Supplementary Table S4-3. Correlation matrix of nasotypes from adult samples of age group 40-64 (N=88) - Spearman r values; Supplementary Table S4-4. Correlation matrix of nasotypes from adult samples of age group >=65 (N=50) - Spearman r values

Supplementary Table S5: All three nasotypes: Significant gene expression changes (p<0.05, FC>1.5) comparing adult patients with asthma aged ≥65 years versus adult patients with asthma aged 18-39 years

Supplementary Table S6: Correlation matrix of nasotypes from all pediatric nasal samples from the URECA cohort (N=353) - Spearman r values

Supplementary Table S7: Correlation matrix of nasotypes from all nasal samples from the GRASS cohort (N=256) - Spearman r values

Supplementary Table S8: Correlation matrix of nasotypes from all nasal samples from the CatEEC cohort (N=256) - Spearman r values

Supplementary Table S9: Trajectory analysis - Driver genes of all different cellular subgroups (terminal states) (Figure 2)

Supplementary Table S10: Trajectory analysis - Driver genes of different E1 cellular subgroups (terminal states) (Figure 2)

Supplementary Table S11: Trajectory analysis - Driver genes of different E2 cellular subgroups (terminal states) (Figure 2)

Supplementary Table S12: Trajectory analysis - Driver genes of different E3 cellular subgroups (terminal states) (Figure 2)

Supplementary Table S13: Significant gene expression changes based on scRNASeq comparing secretory cells of different E-types (Figure 2)

Supplementary Table S14-1: Cluster-to-cell type annotation mapping of cytokine-stimulated NHBE ALI scRNA-seq cultures (Figure 3); Supplementary Table S14-2: Significant marker genes across epithelial scRNA-seq clusters (Figure 3)

Supplementary Table S15: Relative E-state fractions across cytokine stimulation conditions in NHBE ALI cultures (Figure 3)

Supplementary Table S16: Donor-centered E-state expression scores across cytokine stimulation conditions (Figure 3)

Supplementary Table S17: Correlation matrix of nasotypes from healthy pediatric samples (N=79) - Spearman r values

Supplementary Table S18: Correlation matrix of nasotypes from pre-school wheeze pediatric samples (N=81) - Spearman r values

Supplementary Table S19-1. Significant differential expression of E1/E2/E3 nasotype-associated genes in children with preschool wheeze versus healthy (Benjamini-Hochberg adjusted p<0.05, fold change >1.5); Supplementary Table S19-2. Significant differential expression of E1/E2/E3 nasotype-associated genes in children with asthma versus wheeze (Benjamini-Hochberg adjusted p<0.05, fold change >1.5)

Supplementary Table S20-1. Significant differential expression of E1/E2/E3 nasotype-associated genes in children with asthma versus healthy (Benjamini-Hochberg adjusted p<0.05, fold change >1.5); Supplementary Table S20-2. Significant differential expression of E1/E2/E3 nasotype-associated genes in children with asthma versus preschool wheeze (Benjamini-Hochberg adjusted p<0.05, fold change >1.5).

Supplementary Table S21: Correlation matrix of nasotypes from all pediatric samples (N=265) with TAC phenotypes (U-BIOPRED study) - Spearman r values

Supplementary Table S22. Association of age and gene expression of the E1, E2, and E3 index genes: beta coefficient and standard error (SE) based on linear regression

Supplementary Table S23. Association of disease status and gene expression of the E1, E2, and E3 index genes: beta coefficient and standard error (SE) based on linear regression

Supplementary Table S24. Significant differential expression of E1/E2/E3 nasotype-associated genes in healthy children aged 1-6 years versus 13-18 years (Benjamini-Hochberg adjusted p<0.05, fold change >1.5)

Supplementary Table S25. Significant differential expression of E1/E2/E3 nasotype-associated genes in preschool wheezers aged 4-6 years versus 1-3 years (Benjamini-Hochberg adjusted p<0.05, fold change >1.5)

Supplementary Table S26-1: Correlation matrix of nasotypes from children with wheeze or asthma with atopic parameters - Spearman r values; Supplementary Table S26-2: Correlation matrix of nasotypes from children with wheeze or asthma with atopic parameters - Spearman P values

Supplementary Table S27. Subject characteristics of healthy and asthmatic adults of the ALLIANCE cohort (DZL)

Supplementary Table S28. Association of population characteristics and gene expression of the E1 index gene: beta coefficient and standard error (SE) based on linear regression

Supplementary Table S29. Association of population characteristics and gene expression of the E2 index gene: beta coefficient and standard error (SE) based on linear regression.

Supplementary Table S30. Association of population characteristics and gene expression of the E3 index gene: beta coefficient and standard error (SE) based on linear regression.

Supplementary Table S31. Candidate list of epithelial secreted/immune-related transcripts set

**SUPPLEMENTARY REFERENCES**

1.	Zissler UM, Chaker AM, Effner R, et al. Interleukin-4 and interferon-gamma orchestrate an epithelial polarization in the airways. *Mucosal Immunol.* 2016;9(4):917-926.

2.	Chen C, Smith AD, Cheung L, Pham Q, Urban JF, Jr., Dawson HD. Potentiation of IL-4 Signaling by Retinoic Acid in Intestinal Epithelial Cells and Macrophages-Mechanisms and Targets. *Front Immunol.* 2020;11:605.

3.	Hage T, Sebald W, Reinemer P. Crystal structure of the interleukin-4/receptor alpha chain complex reveals a mosaic binding interface. *Cell.* 1999;97(2):271-281.

4.	Wolock SL, Lopez R, Klein AM. Scrublet: Computational Identification of Cell Doublets in Single-Cell Transcriptomic Data. *Cell Syst.* 2019;8(4):281-291 e289.

5.	Wolf FA, Angerer P, Theis FJ. SCANPY: large-scale single-cell gene expression data analysis. *Genome Biol.* 2018;19(1):15.

6.	Hie B, Bryson B, Berger B. Efficient integration of heterogeneous single-cell transcriptomes using Scanorama. *Nat Biotechnol.* 2019;37(6):685-691.

7.	Blondel VD, Guillaume J-L, Lambiotte R, Lefebvre E. Fast unfolding of communities in large networks. *Journal of Statistical Mechanics: Theory and Experiment.* 2008;2008(10).

8.	Traag VA, Waltman L, van Eck NJ. From Louvain to Leiden: guaranteeing well-connected communities. *Sci Rep.* 2019;9(1):5233.

9.	Setty M, Kiseliovas V, Levine J, Gayoso A, Mazutis L, Pe'er D. Characterization of cell fate probabilities in single-cell data with Palantir. *Nat Biotechnol.* 2019;37(4):451-460.

10.	Weiler P, Lange M, Klein M, Pe'er D, Theis F. CellRank 2: unified fate mapping in multiview single-cell data. *Nat Methods.* 2024;21(7):1196-1205.

11.	Altman MC, Calatroni A, Ramratnam S, et al. Endotype of allergic asthma with airway obstruction in urban children. *J Allergy Clin Immunol.* 2021;148(5):1198-1209.

12.	Altman MC, Segnitz RM, Larson D, et al. Nasal and blood transcriptomic pathways underpinning the clinical response to grass pollen immunotherapy. *J Allergy Clin Immunol.* 2023;152(5):1247-1260.

13.	Larson D, Patel P, Salapatek AM, et al. Nasal allergen challenge and environmental exposure chamber challenge: A randomized trial comparing clinical and biological responses to cat allergen. *J Allergy Clin Immunol.* 2020;145(6):1585-1597.

14.	Love MI, Huber W, Anders S. Moderated estimation of fold change and dispersion for RNA-seq data with DESeq2. *Genome Biol.* 2014;15(12):550.

15.	Gu Z. Complex heatmap visualization. *Imeta.* 2022;1(3):e43.
